# Electrophysiological correlates of why humans deviate from rational decision‐making: A registered replication study

**DOI:** 10.1111/psyp.14665

**Published:** 2024-08-13

**Authors:** Johannes Rodrigues, Martin Weiß, Grit Hein, Johannes Hewig

**Affiliations:** ^1^ Department of Psychology V: Differential Psychology, Personality Psychology and Psychological Diagnostics Institute of Psychology, University of Würzburg Würzburg Germany; ^2^ Translational Social Neuroscience Unit, Center of Mental Health, Department of Psychiatry, Psychosomatic and Psychotherapy University of Würzburg Würzburg Germany; ^3^ Department of Psychology I: Clinical Psychology and Psychotherapy Institute of Psychology, University of Würzburg Würzburg Germany

**Keywords:** cognitive control, content/topics, decision‐making, EEG, methods, skin conductance

## Abstract

In contrast to rational choice theory predicting humans to optimize expected utilities of choices, humans deviate from rational behavior in decision‐making paradigms. Hewig et al. (2011) explored affective correlates of decision‐making in the ultimatum game (UG) and the dictator game (DG). They found that feedback‐related negativity (FRN), subjective valence ratings, and autonomic nervous system activity predicted rejection of monetary offers. This registered replication aimed to validate and extend these findings. Although behavioral patterns and results of subjective ratings closely matched the original study, not all psychophysiological effects were successfully replicated. Firstly, we could not replicate the reported effects of autonomic nervous system activity. Secondly, a quadratic instead of the originally proposed linear relation between the offer and the FRN emerged, possibly driven by the offer evaluation in economic games and the rewarding anticipation of successful punishment for low offers. Thirdly, P3 amplitudes mirrored the quadratic offer response pattern, generally peaking for the lowest offer. In contrast to the original study, P3 responses were larger in the UG compared with the DG. Finally, our findings indicate that participant‐related higher midfrontal theta activation predicted lower acceptance behavior in the UG, with a systematic dampening effect for fairer offers. This highlights cognitive control as a crucial mechanism in economic decision‐making to overcome behavioral defaults. Overall, our results conceptually support the original conclusion that decision‐making in economic games is non‐rational and dependent on the objective situation as well as emotional and neural markers, though not precisely as suggested by Hewig et al. (2011).

## Abstract 2

This article presents crucial insights into deviations from rational decision‐making paradigms by replicating and extending Hewig et al.'s (2011) seminal study. While reaffirming key findings, our work unveils novel psychophysiological nuances, advancing understanding of economic game decision‐making, emphasizing its non‐rational nature and the role of cognitive control.

## 1 INTRODUCTION

For many years, psychologists and economists have conducted experiments with bargaining games to model real‐life negotiation situations in the laboratory. A negotiation situation such as a contract or other business negotiation can be modeled with a tool called the ultimatum game (UG; Güth et al., 1982). This game is played by two people, with one person (the proposer) suggesting a way to split a stake between themselves and the other person (the responder). If the responder accepts this offer, the stake is split in the proposed way. However, if the responder rejects it, neither receives a share of the stake. As noted by Güth et al. (1982), each individual participating in the UG essentially acts independently in his or her own interest. The UG measures how much a proposer will offer or how little a responder will accept depending on the circumstances. This enables the researcher to examine how these behaviors interact with strategy formation (Güth & Kocher, 2014).

An advancement of the UG resulted in a second type of bargaining game, the dictator game (DG). It was introduced by Kahneman et al. (1986) to model charitable giving and thus examine behavior related to other persons' as opposed to one's own interests. Today, experiments commonly use a simplified version of the DG developed by Forsythe et al. (1994) (Engel, 2011). In this two‐person game, the proposer (dictator) also offers a portion of his or her stake to the responder. However, in contrast to the UG, the responder cannot reject the proposal.

Although some individuals act rationally and utility‐maximizing in both paradigms, there are others who propose (UG) or give away (DG) money, but reject unfair offers as responders in the UG, even if they deviate only slightly from a fair distribution (e.g., Camerer, 2003). Research shows that beyond strategic considerations, certain emotional states can lead people to deviate from rational decisions. This emphasizes the importance of effect in economic decision‐making, particularly regarding the UG responder behavior: Although anger (Pillutla & Murnighan, 1996), sadness (Harlé & Sanfey, 2007), and disgust (Moretti & di Pellegrino, 2010) were previously shown to increase rejection rates, induced happiness was related to an increase in acceptance rates (Riepl et al., 2016). Other research revealed that negative mood seems to increase concerns about the fairness of allocations, whereas positive mood decreases such concerns (Forgas & Tan, 2013) and alleged social observations raised rejection rates, possibly due to implicit norm reinforcement (Peterburs et al., 2017).

Previous neuroscientific studies have used electroencephalography (EEG) and event‐related potentials (ERPs) to investigate brain activity in response to fair and unfair offers in bargaining games. Both in the UG (e.g., Boksem & de Cremer, 2010; Polezzi et al., 2008) and in the DG (e.g., Wu et al., 2011), an indicator of medio‐frontal negativity, the feedback‐related negativity (FRN), was more pronounced in unfair compared with fair offers. The FRN is typically measured in response to performance feedback (e.g., Holroyd & Coles, 2002) and occurs approximately 200–400 ms after stimulus onset (Holroyd et al., 2008). It peaks around 240–320 ms after stimulus onset (e.g., Miltner et al., 1997) and reflects activity related to evaluative processes in the anterior cingulate cortex (ACC; Boksem & de Cremer, 2010; Rodrigues et al., 2020, 2022).

Other researchers have focused on the P3 component, a later relative positivity in the ERP, which originates from temporal–parietal regions. It has been linked to attentional processes (e.g., Polich, 2007), stimulus evaluation, and decision processes in general (Duncan‐Johnson & Donchin, 1982; Karis et al., 1983). Moreover, P3 has been associated with later integrative stages of feedback processing that allow for coding of outcome ambivalence (e.g., Peterburs et al., 2013) and fairness (e.g., Riepl et al., 2016).

In pursuit of fostering reproducibility in neuroscience, we aimed to replicate a seminal study investigating affective correlates of social decision‐making. Since the same conceptual framework and method are used, the documentation of replication studies is intended to reveal all differences and similarities with the original study (Brandt et al., 2014). Therefore, this manuscript briefly reports the purpose of the original study and the replicated method while also explaining specific extensions. These were implemented to improve methodological shortcomings (e.g., sample size) or analytical shortcomings (e.g., new EEG‐analysis pipelines or analysis at single‐trial level) of the original study. A full report of the conceptual background and method of the original study can be found in Hewig et al. (2011).

### 1.1 Why the to‐be‐replicated result holds empirical and theoretical significance

The objective of the study conducted by Hewig et al. (2011) was to investigate basic mechanisms and individual differences in social decision‐making using one‐shot versions of the UG and DG. More specifically, the authors aimed to unravel affective processing in these paradigms on multiple levels. To achieve this, the authors assessed decision‐making behavior, subjectively perceived affect, and activity related to the central and autonomic nervous system as quantified via EEG signals and skin conductance response (SCR), respectively. The central hypothesis was that subjective negative affect, larger SCR, and larger (more negative) FRN amplitudes as indicators of negative affective processing would be related to larger deviations from “rational decisions” in accordance with the homo economicus and rational choice theory (von Neumann & Morgenstern, 1944). The hypothesis postulated and confirmed by Hewig et al. (2011) suggests a unified affective processing of fairness spanning from subjective perception to the autonomic and central nervous system and has since contributed to a large body of subsequent influential research across different domains (Campanhã et al., 2011; Karagonlar & Kuhlman, 2013; Mussel et al., 2013; Wu et al., 2013).

### 1.2 Why replicability cannot be considered well‐established for this specific finding

To the best of our knowledge and despite the preceding evidence on which Hewig et al. (2011) have built their work (Polezzi et al., 2008; van ’t Wout et al., 2006), no close or direct replication of the results reported by Hewig et al. (2011) has yet been undertaken to test the consistency of the role of perception, SCR, and FRN with respect to affective processing of fairness‐related rational choice behavior. Moreover, the study results of Hewig et al. (2011) are based on a sample of 12 participants (eight males and four females). This raises concerns about the statistical power of the study, especially as some non‐significant findings may be disproportionately influenced by the rather small sample size. In addition, other measures for the processes underlying behavioral decisions have since been established and can now be analyzed in this context, such as midfrontal theta (MFT) activity and single‐trial EEG responses (see “1.4 Extensions of the replication”).

### 1.3 Criteria describing a successful or failed replication

The planned endeavor will be considered a successful replication if we can show

- that more unfair offers lead to more rejection of the offers,
- “smaller” (more positive) FRN amplitudes in response to fair offers,
- higher P3 responses to the most unfair and the most fair offers,
- that more negative emotional ratings predict higher rejection rates,
- that larger SCRs predict higher rejection rates, and
- that taken together, FRN amplitudes to fair offers, SCR amplitude, and emotional valence ratings account for ≥50% of variance in individual differences in rejection rates for the 10:2 offer.

The findings above are the main targets of this replication and should be independent of EEG data processing based on the original study compared with a standardized EEG pipeline (Rodrigues et al., 2021). The (full) replication was considered failed if any of the six analyses (a–f) failed to demonstrate a significant effect of the offer on the respective outcome. In addition, the replication was also considered failed if the FRN effects were absent or even reversed after applying a standardized method to preprocess the EEG data.

### 1.4 Extensions of the replication

Differences between the present and the original study include a new research setting, new research assistants, and a different university. The original study's images of human faces were replaced by a new set of stimuli to ensure timeliness of the study to the participants. Moreover, we recruited a larger sample for the replication and adapted the presentation times of the stimuli (see “2.3 Task and procedure”). In the data analysis, apart from the classical analysis of variance (ANOVA) for the FRN with the factors experiment (UG vs. DG) and offer, additional analyses were performed at the single‐trial level.

Furthermore, we added MFT activation to our analyses as the neural response representing the evaluation of outcomes and outcome expectancy is not only associated with the FRN (Holroyd & Coles, 2002) but also with MFT activation (e.g., van der Molen et al., 2017). Although some findings have suggested similar functioning of MFT and FRN (e.g., Rodrigues et al., 2015), more recent research demonstrated MFT as a significant predictor of decreasing third‐party punishment in a third‐party DG (Rodrigues et al., 2020) and of behavioral changes following direct feedback of responder's behavior in the UG (Bogdan et al., 2022). One possible explanation could be that MFT activation, as an indicator of cognitive control (Cavanagh & Frank, 2014; Cohen, 2011), represents the overcoming of a default behavioral tendency (Rodrigues et al., 2020, 2022). In our analyses, we performed additional analyses to compare the predictive power of the FRN with that of MFT at the single‐trial level. Given recent evidence explained below, we expected that both electrocortical markers would be linked to the offer size in the UG (e.g., Mothes et al., 2016), but that MFT would be a better predictor of the rejection of unfair offers. In DG, however, the FRN should be linked to the fairness of the offer, whereas MFT should not be linked to the offer. This is because no behavioral response is required, and consequently, there is no need to control for the behavioral default of acceptance (Rodrigues et al., 2022). This assumption was based on a study comparing the standard UG with a two‐stage version, where the second stage only appears after a rejection in the first stage. In the normal UG, a more negative FRN and higher MFT were linked to lower UG offers. However, in the two‐stage UG, the MFT was inversely associated with lower offers in the first stage, whereas the FRN continued to relate to lower offers. The authors concluded that the FRN was associated with the expectance evaluation concerning fairness, whereas MFT marked the cognitive control required to overcome the default behavior of accepting an offer in the normal UG (higher MFT in case of a lower offer) and the default behavior of rejecting the first offer in the two‐stage UG (higher MFT in case of a high offer). Following this interpretation and given the rejection option in the normal UG, the MFT should predict the rejection behavior rather than the fairness response alone.

These analytical extensions did not affect the experimental design of the original study. However, the original study was unable to explore possible differences in magnitudes of the FRN and MFT responses to unfair offers in the different types of games due to the small sample size and study design. The DG provides no opportunity for interacting with the proposer, or in that case the dictator, potentially leading the receiver to expect worse offers compared with the UG (Haselhuhn & Mellers, 2005; Mellers et al., 2010). Accordingly, compared with the UG, the FRN response in the DG is expected to be dampened, especially for offers that are not entirely unfair. More repetitions of the DG than in the previous version are needed to adequately compare DG and UG. Moreover, the order of the DG and UG must be randomized. Finally, there have been significant developments in EEG (pre)processing in the last 10 years. Consequently, beyond replication, a standardized EEG (pre)processing pipeline will be used to ensure objectivity in artifact correction (Rodrigues et al., 2021).

## 2 METHOD

### 2.1 Ethics statement

The study was carried out in accordance with the recommendations of the “Ethical Guidelines” of “The Association of German Professional Psychologists” (“Berufsethische Richtlinien, Berufsverband Deutscher Psychologinnen und Psychologen”). All participants provided written informed consent in accordance with the Declaration of Helsinki before participating in the experiment. The protocol was approved by the local ethics committee of the Department of Pychology of the Julius‐Maximilians‐University of Würzburg, Germany (Ethikkommission des Institutes für Psychologie der Humanwissenschaftlichen Fakultät der Julius‐Maximilians‐Universität Würzburg).

### 2.2 Sample

We recruited participants via postings on the internet and within buildings of the University of Würzburg. All participants were offered course credit (for students of Psychology) or a monetary compensation equal to the German minimum wage (~12 € per hour). The monetary compensation consisted of a general participation compensation of ~6 € and a bonus win of ~10–12 €, similar to the procedure used by Hewig et al. (2011).

An a priori estimate of the required sample size was conducted in *R* using the “Superpower” package by Lakens and Caldwell (2021). The complexity of our interaction in the Generalized Linear Model (GLM; 6*5*3*2) could not be computed as only 3 factors are supported. Therefore, we calculated the sample size based on a 6*5*3 design, omitting the comparison of the UG with the DG. We used the original data of Hewig et al. (2011) as effect estimation. Their sample size was also used as an estimate for the multilevel models control concerning the power. Due to the limitations of the software, a sample size smaller than the product of the factors is not viable. With the recommended sample size of *n* = 91 and the two matrices for the UG or DG (see below), the estimated power for every main effect and interaction using the respective model (see Table 1) exceeds 90. This takes into account an estimated correlation of the different conditions of 0.9, a cautious estimate derived from the original physiological data, and homogeneity of the measurements of *α*_Cronbach_ = .99.

**TABLE 1:** Estimated power of interaction based on effect sizes, using data simulation with the Superpower package and the original data of Hewig et al. (2011).

	Power	Partial_eta_squared	Cohen_f	Non_centrality
A	100	0.94	3.95	5611.18
B	91.23	0.07	0.27	13.42
C	100	0.80	1.98	1772.75
a:b	100	0.15	0.43	131.59
a:c	100	0.30	0.65	768.25
b:c	100	0.12	0.36	118.81
a:b:c	100	0.04	0.21	165.37

**FIGURE 1:**
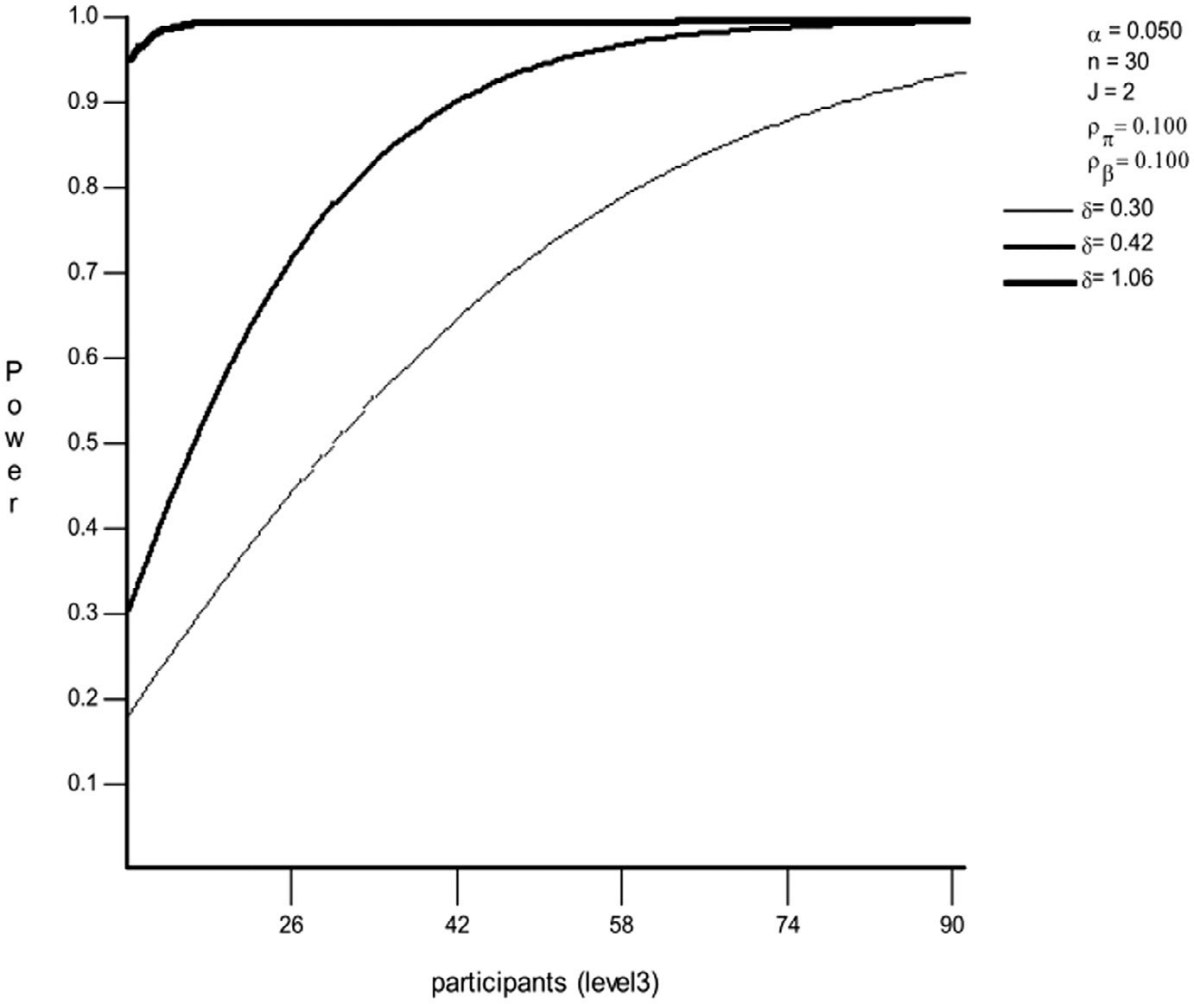
Power estimate of the three‐level model, based on the estimated effect size delta, Level 2 (2), and the number of trials on Level 1 (30). Graphics created using Optimal design.

The optimal design (Raudenbush et al., 2011) power calculation for the estimated sample size of 91 participants is above a power of 0.90 for every effect estimate used in this case (see Figure 1). The estimates were based on the smallest significant given effects in the original study (*δ* = 1.06) and the original data in the design, estimated with the Superpower package even when no effect was found (Cohen's *f* = .21, δ = .42), and calculated for the effect size *f* = 0.15 as an even further cautious estimate for the interactions in future data (*δ* = .3).

We collected data from 107 participants, accounting for an expected 10% data loss due to recording errors and expected bad signal quality. Only two participants had to be excluded (one because of data recording error and one because of aberrant behavior, that is, possibly a misunderstanding what was displayed in what fashion on the pc, as the behavioral responses were exactly opposite to the expected and well‐known behavioral patterns), leading to a final sample of 105 participants (mean age = 26.12, SD = 8.82, range = 18–65; gender: 35.25% male, 63.81% female, 0.95% diverse; handedness: 86.66% right, 12.28% left, 0.95% ambidexter). Exclusion criteria were mental illnesses (0), recording errors (1), deep knowledge about the paradigm and the cover story (0), or classification as outlier if *z* > 3.29 (Tabachnick & Fidell, 2007) for behavioral responses (1). To assess whether the cover story had worked and whether the participants knew too much about the paradigm, we asked the participants how confident they were that the information given about the experiment was correct and whether they knew about the paradigm and its aim (for details, see the section below).

### 2.3 Task and procedure

Participants were seated individually in a dimly lit and temperature‐controlled EEG cabin, and an EEG cap and SCR electrodes were applied (see Section 2.3.1). Each participant played both the UG and the DG in a series of one‐shot trials. To enhance the credibility of the cover story (see below), the participants first briefly played both paradigms as proposers. As in the original study, 40 proposer trials were conducted in the UG and 10 in the DG. In each trial as a proposer, the participants were instructed to divide a fixed amount of money (12 cents) into two shares: one for him‐ or herself and the other for the responder. Participants were informed that their proposal would be stored and used for future participants. They were also informed that, in their subsequent role as responders, they would receive only one offer from a specific proposer. To further increase the plausibility of the cover story, a photograph was taken of each participant. These pictures, along with those of the virtual proposers, were presented following the feedback about the monetary outcome of each trial in the responder games.

After playing the game as proposers, participants were prepared for the EEG and SCR recordings (see Section 2.3.1). Then, the participants played the games in the role of the responder. They were presented with a randomized series of 180 offers (30 for each of 6 conditions: 6:6, 5:7, 4:8, 3:9, 2:10, and 1:11) for UG and DG, respectively (original study: 240 trials of UG and 60 trials of DG), resulting in 360 trials divided into six blocks (i.e., 60 trials per block) with at least 10 s of breaks between each block. The adjustments in the trial repetitions were made to ensure a balance between UG and DG trials and to prevent excessive participant boredom. All changes made to the original version of the paradigms are listed in Table 2. In contrast to the original study where all trials were presented without breaks, the participants initiated each new block in the present setup. Participants had the option to either accept or reject any proposal. If accepted, the money was divided according to the proposal; if rejected, neither the proposer nor the responder received anything. In the original study, there was no randomization between participants for the acceptance and rejection keys. Here, we randomized the acceptance and rejection keys between participants. As responders in the DG, participants could not respond and thus “accepted” any offer automatically. However, a change to the previous study was implemented, requiring the participants to press one of the two response buttons (i.e., left or right arrow key) to proceed to the feedback. Participants were explicitly informed that there was no difference between using the left or the right arrow key. This modification was added to enhance comparability and reduce confounding factors between the DG and UG, as both now required a button press. In both games, participants were instructed to pay attention to the screen; however, in the UG, they could make a decision, while in the DG, they simply pressed the button to complete the trial. The blocks of the UG and DG were presented consecutively, but the order was randomized between participants. During each block, each trial started with the presentation of a fixation cross (750 ms in the original study, now jittered between 300 and 500 ms). As in the original study, this was followed by an indication of the offer of the (fake) proposer via a divided color bar in a 2400 ms response window. The right‐sided portion in blue indicated the amount offered by the proposer, whereas the left‐sided portion in red indicated the amount retained by the proposer. Although it would have been preferable, the colors of the proportions were not randomized in the original, a choice we adopted for replicational purposes. In the original study, after 400 ms, a tone (100 ms duration, 800 Hz) prompted the participants to respond by either accepting or rejecting the offer within 2 s. However, based on evidence showing that (task‐irrelevant) SCR is affected by tones (e.g., Frith & Allen, 1983; Vrana, 1995), we anticipated that the tone could potentially bias and distort the valence‐based responses in the EEG and especially the SCR. Consequently, we omitted the tone from the replication study. Immediately after the participant's button press response, the amount of money resulting from their decision in the corresponding trial was presented for 600 ms. Finally, photographs of the participant and the fake proposer in that trial were displayed for 1 s, along with the amount of money each had received and the cumulative amount of the participant's winnings. In each trial, a different proposer was randomly selected from a set of photographs, either taken from preceding participants with their respective consent or from an archive of face images. No proposer image was presented twice throughout the experiment. After having completed all blocks of a game type, we assessed participants' subjective ratings about the emotional valence and arousal of each offer in each condition on a 9‐point rating scale (ranging from 1 = “very negative” to 9 = “very positive” or from 1 = “very calm” to 9 = “very arousing”).

**TABLE 2:** Design differences between Hewig et al. (2011) and the replication study and analytical steps exceeding the pure replication. A rationale for design deviations and extensions can be found in brackets or the method section.

	Hewig et al. (2011)	Replication and extension
Design changes		
Recruitment site	Jena (Germany)	Würzburg (Germany)
Task order	First UG, then DG	Randomized between participants
UG trials	240 (40 for each of 6 offers)	180 (30 for each of 6 offers)
DG trials	60 (10 for each of 6 offers)	180 (30 for each of 6 offers)
Fixation cross duration	700 ms	400 ± 100 ms (to avoid anticipation effects due to fixed interval)
UG response	Tone + left/right arrow key	Left/right arrow key
DG response	None	Left/right arrow key to complete trial
UG response	Acceptance/rejection key order fixed	Acceptance/rejection key order counterbalanced between participants
EEG montage	128‐channel electrode cap	66‐channel electrode cap
SCR recording	Foot	Hand (to avoid too low effects due to lower reactivity of the foot)
SCR electrodes	6 mm diameter and 0.28 cm^2^ recording area	7 mm diameter and 38.48 mm^2^ recording area
Rating scales	Valence	Valence and arousal
Extended analysis		
Sample size	*N* = 12	*N* = 105
EEG (pre‐)processing	State of the art procedure at the time	Standardized and automated pipeline (Rodrigues et al., 2021)
EEG offline reference	Linked mastoid	Current source density, linked mastoid
Neural correlates	FRN, P3	FRN, MFT, P3
FRN/MFT electrode site	Fz	FCz
Time windows	FRN: 280–320 ms	FRN/MFT: 250–550 ms
	P3: 350–450 ms	P3: 300 ms–700 ms
Peak quantification	Average across whole time window	FRN/MFT: ±40 ms around peak
		P3: ±100 ms around peak
SCR filter	0.1 Hz high‐pass filter	0.05–2 Hz bandpass filter
SCR measurement	Maximum skin conductance (only positive values)	Area bounded by the SCR curve
Analyses	ANOVAs and multiple regressions	Linear mixed models at the single‐trial level

Abbreviations: DG, dictator game; EEG, electroencephalography; FRN, feedback‐related negativity; MFT, midfrontal theta; SCR, skin conductance response; UG, ultimatum game.

#### 2.3.1 EEG and skin conductance recording and quantification

EEG and SCRs were recorded while participants assumed the role of the responder. Ag/AgCl electrodes were applied for the measurement of EEG. Although the original study used a 128‐channel montage and an electro‐oculogram (EOG), we applied a 66‐electrode cap (according to the international 10–10 system) without additional EOG channels. As our data analysis chain used independent component analysis (ICA) for eye‐artifact correction (see EPOS, Rodrigues et al., 2021), we aimed to use a cap montage, which maximizes EEG sources to obtain as many usable EEG signals for the ICA‐matrix as possible. Unfortunately, we lacked the option to obtain caps with 128 electrodes exclusively for this study. However, using ICA, a higher density of 64 sources is seldomly employed, whereas with 128 electrodes, principal components analyses (PCA) were commonly used in previous research (see e.g., Gabard‐Durnam et al., 2018; Miyakoshi, 2021; Rodrigues et al., 2021). The online reference was vertex (Cz), whereas the offline reference was linked mastoids (similar to the original study). All impedances of electrodes were kept below 5 kΩ, and the differences of impedance between homologous sites were kept below 1 kΩ. EEG was amplified using two 32‐channel DC BrainAmp MR plus amplifiers (BrainProducts, Munich, Germany; input impedance: 10 MO). The sampling rate was 500 Hz and the bandpass filter was set to 0.015–250 Hz. For preprocessing, the processing steps of the EPOS pipeline (Rodrigues et al., 2021) were executed using MATLAB and EEGlab (Delorme & Makeig, 2004). For channel extrapolation and segment selection, the *z*‐value criterion of *z* = 3.29 (Tabachnick & Fidell, 2007) was used. This led to a mean interpolation of m = 6.53 channels (SD = 2.01) and a mean rejection of m = 4.68 segments (SD = 4.23). For the selection of artifact ICs in ICA, MARA (Winkler et al., 2011) and ADJUST (Mognon et al., 2011) were used in SASICA (Chaumon et al., 2015). This led to a mean rejection of artifact segments (e.g., eye artifacts, pulse artifacts, movement artifacts, muscle artifacts, and line noise) of m = 39.23 ICs (SD = 6.67). The segmentation was done from 150 to 1400 ms following stimulus onset (presentation of the offer distribution), with a baseline interval from −100 to 0 ms. A matching peak for FRN was detected after applying a 40 Hz Butterworth low‐pass filter via the time course of the ERPs on the electrode position FCz (Fz in the original study) in the time window from 250 to 550 ms for the mean signal of all conditions and participants. For direct replication, we first analyzed the average peak amplitude in the time windows 280–320 ms (FRN at electrode site Fz) and 350–450 ms (P3 at electrode site Pz) for each participant and each experimental condition. As an extension, an alternative FRN time window for the mean signal of all conditions and participants was detected at electrode position FCz in the time window 250–550 ms by wrapping a 40 ms window around this peak. An alternative P3 time window was identified by detecting the positive peak of the mean signal at Pz in the time window 300–700 ms (original study: 350–450 ms) and wrapping a 100 ms window around this peak.

Skin conductance was recorded from the caps of the left index and middle finger (Figner & Murphy, 2011, original study: the sole of the left foot) using Ag/AgCl electrodes (diameter of contact area between skin and electrode paste: 7 mm diameter, recording area = 38.482 mm^2^; original study: 6 mm diameter, recording area = 0.28 cm^2^) and the Brainvision ExG Amplifier. Based on previous research (Payne et al., 2013) and our own experience indicating that SCR recorded from fingers is more responsive than SCR recorded from feet, we chose to diverge from the original study and record SCR from fingers instead of feet. The electrodes were filled with TD‐246 Skin Conductance Electrode Paste (0.5% saline in neutral base, Discount Disposables, St. Albans, Vermont). The skin conductance signal was filtered with a 0.05–2 Hz MATLAB bandpass filter in order to correct for high‐frequency noise like light or electrical signal noise from the monitor (Figner & Murphy, 2011; original study high‐pass filtered 0.1 Hz). Baseline correction was applied for −1000 to 0 ms pre‐stimulus. The skin conductance measurement was obtained by using the area bounded by the SCR curve (Figner & Murphy, 2011), with a window of interest from 1.5 to 7.5 s after onset of the offers (following the recommendation of a measurement window about 5–6 s, with an onset between 1 and 3 s after stimulus onset, c.f. Boucsein et al., 2012; Figner & Murphy, 2011). The original study had observed maximum skin conductance in the same time window, but positive values only. The quantification of the area under the curve was calculated by the integral under the SCR curve and a sloped line delineated by the intersection of the measurement window (c.f. method from Naqvi & Benchara (2006) in Figner & Murphy, 2011). Then, the integral was standardized by the length of the time window to obtain μS^2^/second. This approach was chosen due to its higher sensitivity and capacity for automatization compared with the phasic peak measurements employed previously (Figner & Murphy, 2011). Although less commonly used, this method combines the measurement of the amplitude peak with the duration of the response (Boucsein et al., 2012), thus allowing for a quantification of the response magnitude.

#### 2.3.2 Extension

In addition to the measures mentioned above, we used Current Source Density transformation of the EEG signal (CSD; e.g., Kayser, 2009; Kayser et al., 2010) to achieve an additional offline reference. CSD transformation is applied using the toolbox provided by Delorme in EEGlab.

MFT activation at electrode position FCz was also quantified. We extracted theta frequency from 4 to 8 Hz using Morlet wavelets during the event period to see the temporal function of theta. The theta peaks were detected via automatic peak detection for the mean frequency response across all conditions at the FCz electrode, within the same time window as the FRN (250–550 ms).

### 2.4 Statistical analyses

All statistical analyses were carried out using JAMOVI (The jamovi project, 2023) and *R* (R Core Team, 2023) with the packages “glmmTMB” (Brooks et al., 2017) for multilevel modeling and “afex” (Singmann et al., 2019) for ANOVAs. For graphical illustration, the package “ggplot2” (Wickham, 2016) was used.

#### 2.4.1 Replication analyses

To replicate the statistical analyses as close as possible, a 6 × 2 within ANOVA with the within factors fairness (six levels from 6:6: to 11:1) and game (UG vs. DG) was computed for SCR as dependent variable.

For the analyses of FRN and P3 as dependent variables, 6 × 2 × 5 × 3 ANOVAs (original study: 6 × 2 × 5 × 5 ANOVA) were calculated using the mastoid‐referenced values. The within factors were fairness (six levels from 6:6: to 11:1), game (UG vs. DG), and the two topographical factors anteriority (five levels: frontal, frontocentral, central, centroparietal, and parietal) and laterality (three levels: left, midline, and right). For the topographical factors, the following 15 channels were used: F3, Fz, F4, FC3, FCz, FC4, C3, Cz, C4, CP3, CPz, CP4, P3, Pz, and P4 (the original study additionally included the electrode positions F1, F2, FC1, FC2, C1, C2, CP1, CP2, P1, and P2). Concerning the affective ratings, a 6 × 2 within‐subjects ANOVA with the within factors fairness (six levels from 6:6: to 11:1) and Game (UG vs. DG) was computed. Finally, to analyze the rejection behavior in the UG, a simple within‐subjects ANOVA with the factor fairness (six levels from 6:6: to 11:1) was computed for the times of rejection.

In addition, we conducted a stepwise multiple regression with rejection rates for the 10:2 offer as dependent variable. First, we included only emotional valence ratings into the model (Model a). Next, we added SCR amplitudes as predictor (Model b) before finally including FRN amplitudes to fair (6:6) offers (Model c).

#### 2.4.2 Extended analyses

As an extension of the previous work, we conducted the analysis of the CSD‐referenced EEG signal using the same analysis approach as for the linked mastoid reference. Furthermore, a single‐trial analysis (see Albrecht & Bellebaum, 2021; Rodrigues et al., 2020, 2022) concerning the FRN and MFT responses at the electrode position FCz as well as P3 responses at Pz was computed using multilevel mixed models with a random intercept for each participant. The fixed effect offer was inserted at Level 1, the fixed effect of the game (UG vs. DG) at Level 2, and the participants constituted the cluster for Level 3. The metric predictors on Level 1 were centered within participants and games, whereas the variables on Level 3 were grand‐mean centered. The best model was chosen using the corrected Akaike information criterion (AICc) and the probability of information loss (Burnham & Anderson, 2002).

We further assessed the predictive value of the EEG signals for behavioral responses in the UG using a hierarchical single‐trial, multilevel logistic regression model. The fixed effects of offer and the EEG signals FRN per trial, P3 per trial, and MFT per trial were inserted on Level 1. The participants were the cluster variable constituting Level 2. On Level 2, the fixed effects of the mean FRN activation per person, mean P3 activation per person, and mean MFT activation per person were entered. The metric predictors on Level 1 were centered within the participants, whereas the variables on Level 2 were grand‐mean centered. A random intercept for each person was added to the model to account for differences in behavioral preference. Additionally, a fixed effect for the position of the acceptance button (left/right) was exploratorily added to the previously best‐fitting model to examine whether this control variable explained additional variance. The valence ratings were also added to the models, mirroring the original work of Hewig et al. (2011). The best model was again chosen using the AICc and the probability of information loss (Burnham & Anderson, 2002). All post‐hoc *t* tests were Bonferroni‐Holm corrected.

To explore the test–retest reliability of the SCR, the intraclass correlation coefficient (ICC; 2, k) (Koo & Li, 2016) was computed using the “simply agree” package (Caldwell, 2022). To explore the variance explained by the MLMs, we computed *R* ^2^_conditional_ using the random effects and the fixed effects as well as *R* ^2^_marginal_ using only the fixed effects (Nakagawa & Schielzeth, 2013) with the “performance” package (Lüdecke et al., 2023).

## 3 RESULTS

### 3.1 Behavior

**FIGURE 2:**
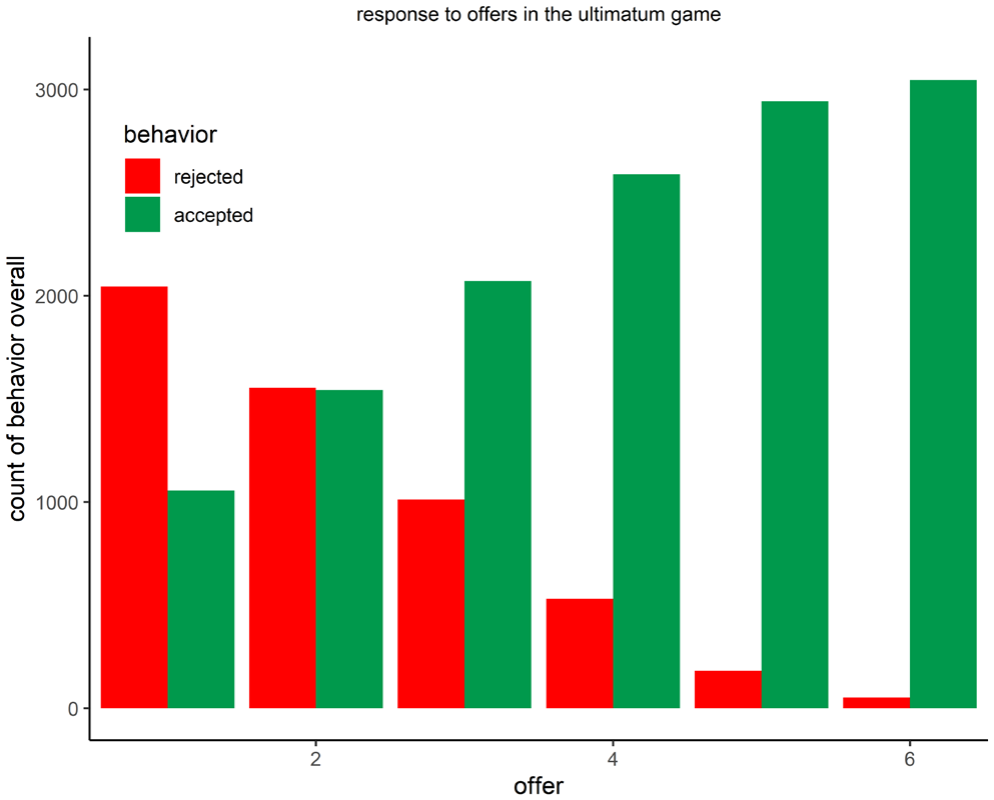
Acceptance dependent on offer.

The ANOVA for the mean reject / acceptance behavior (reject = 0, acceptance = 1) led to a significant effect for offer fairness (*F*(5,520) = 128.06, *p* < .001, $\eta_{p}^{2}$ = 0.55, GG = 0.47), indicating a gradient where the most unfair offers were rejected most frequently and the least unfair offers were rejected least often (*p*s < .05; see Figure 2). This is in line with target (a) of the replication, indicating that more unfair offers lead to more rejection of the offers.

### 3.2 EEG results

The results of the FRN and P3 analyses are reported below, starting with the FRN results and distinguishing between the analyses for the replication, the extension using the individualized time windows (extension_time‐window_), the extension using the CSD reference (extension_CSD_), the extension using single‐trial analysis (extension_single‐trial_), and the MFT‐analysis as an additional neural correlate beyond the replication.

#### 3.2.1 FRN

##### Offer

In the replication analysis, the main effect of offer (*F*(5,520) = 8.73, *p* < .001, $\eta_{p}^{2}$ = 0.08, GG = 0.86) revealed a smaller (more positive) FRN for the lowest offer compared with all other offers (*p*s < .001). The second‐lowest offer was less negative than the two middle offers of three and four credits (*p*s < .05), and the highest offer of six credits was less negative than the offers of five, four, and three credits (*p*s < .001).

**FIGURE 3:**
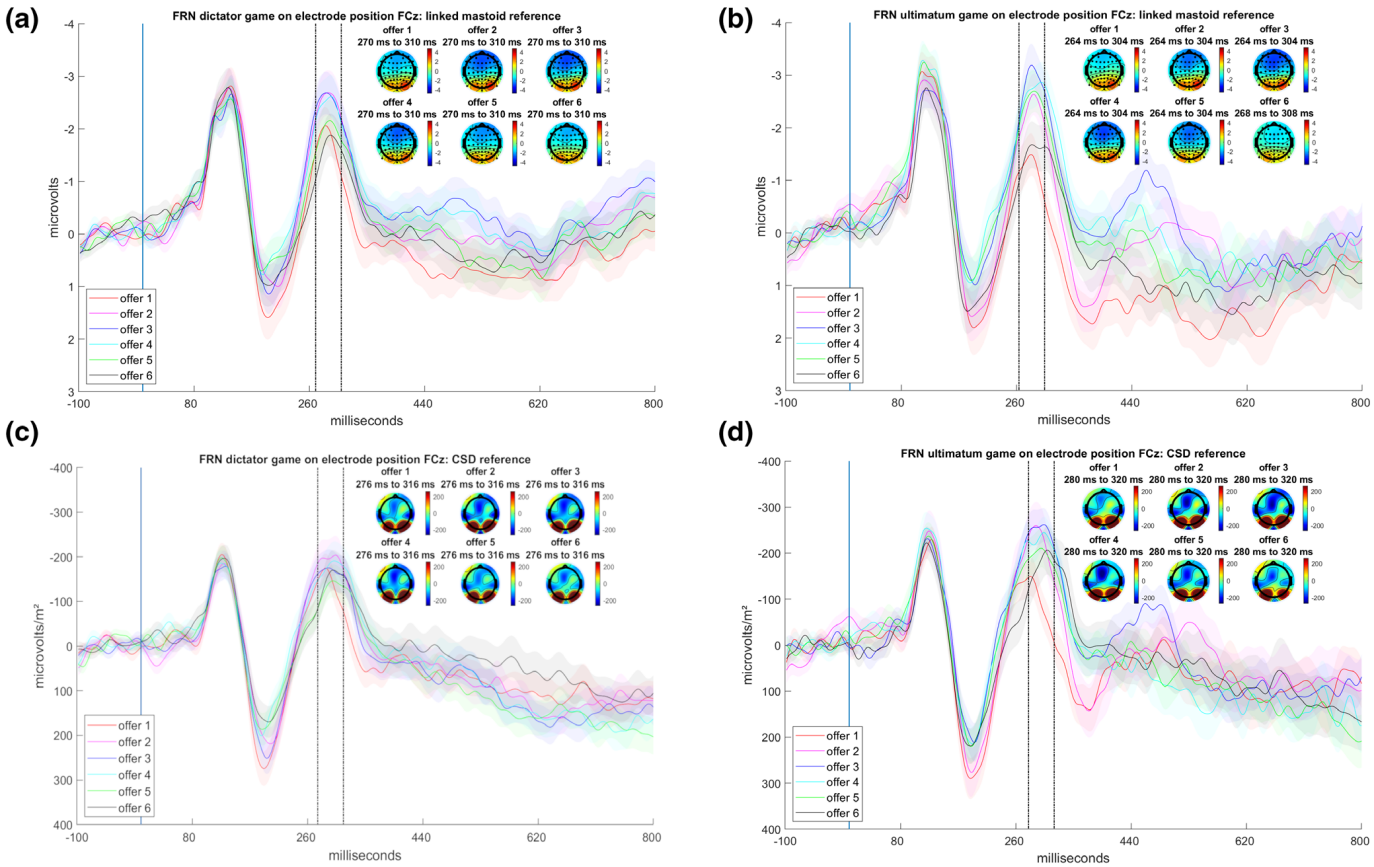
(a) Feedback‐related negativity (FRN) response to the offers in the dictator game (DG) with linked mastoid reference. (b) FRN response to the offers in the ultimatum game with linked mastoid reference. (c) FRN response to the offers in the DG with CSD reference. (d) FRN response to the offers in the ultimatum game with CSD reference. Shaded error bars display the between SEM.

In the extension_time‐window_ analysis, the main effect of offer (*F*(5,520) = 7.31, *p* < .001, $\eta_{p}^{2}$ = 0.07, GG = 0.89) indicated a smaller (more positive) FRN for the lowest offer compared with all other offers (*p*s < .001), whereas the highest offer had the second‐smallest FRN (*p*s < .05). Also, the second‐lowest offer was less negative than the three‐credit offer (*p* < .01). A graphical illustration of these effects is found in Figure 3a,b.

In the extension_CSD_ analysis, the main effect of offer (*F*(5,520) = 7.16, *p* < .001, $\eta_{p}^{2}$ = 0.06, GG = 0.84) measured across all electrodes revealed a more positive FRN for the lowest offer compared with the two highest offers (*p*s < .05). A graphical illustration is found in Figure 3c,d.

**FIGURE 4:**
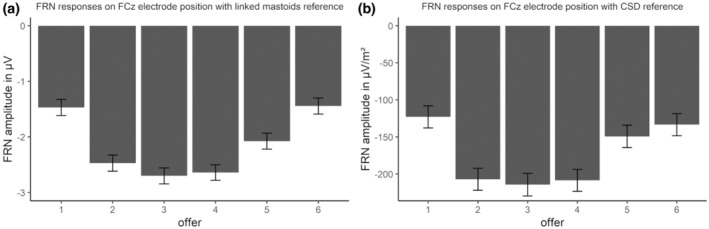
(a) Feedback‐related negativity (FRN) response for linked mastoids reference on FCz. (b) FRN response for CSD reference on FCz. Error bars depict the mean within SEM.

In the extension_single‐trial_ analysis, the best‐fitting model for the linked mastoid‐referenced data with the new peak‐time window quantification as well as the CSD data with the new peak‐time window quantification was the model that only accounted for the offer and not the paradigm (see Table S1). For linked mastoids (see Figure 4a and Table S2), the offer effects indicated a U‐shaped effect, with the lowest offer having more positive FRN values than the others (*p*s < .05), except for the highest offer (*p* = 1), which also exhibited higher values than all other offers (*p*s < .01). In addition, the five‐credit offer had more positive FRN values than the three‐ and four‐credit offers (*p*s < .05). Similarly, for CSD (see Figure 4b and Tables S1 and S2), the lowest offer had more positive FRN values than the rest (*p*s < .001), with the exception of the highest and second highest offer (*p*s > .726), which also showed higher values than the other offers (*p*s < .05).

In summary, the offer effects do not support target (b) of the replication, which expected smaller (more positive) FRN amplitudes in response to fair offers.

The details concerning anteriority, laterality, and their interactions with offer and paradigm are mentioned in the Supplemental Materials S11.

#### 3.2.2 P3

##### Offer

The P3 results revealed a significant main effect of offer for all three analyses (replication: *F*(5,520) = 10.14, *p* < .001, $\eta_{p}^{2}$ = 0.09, GG = 0.88; extension_time‐window_: *F*(5,520) = 8.39, *p* < .001, $\eta_{p}^{2}$ = 0.08, GG = 0.84; extension_CSD_: *F*(5,520) = 17.84, *p* < .001, $\eta_{p}^{2}$ = 0.15, GG = 0.83). Post hoc tests in the replication analysis indicated the highest P3 amplitude for the lowest offer (*p*s < .001), followed by the second‐lowest and the highest offer (*p*s < .05), which did not differ from each other (*p* = .752), and then the five‐credit offer (*p*s < .05). The lowest P3 was found for the middle offers of three and four credits, which did not differ from each other (*p* = .752).

**FIGURE 5:**
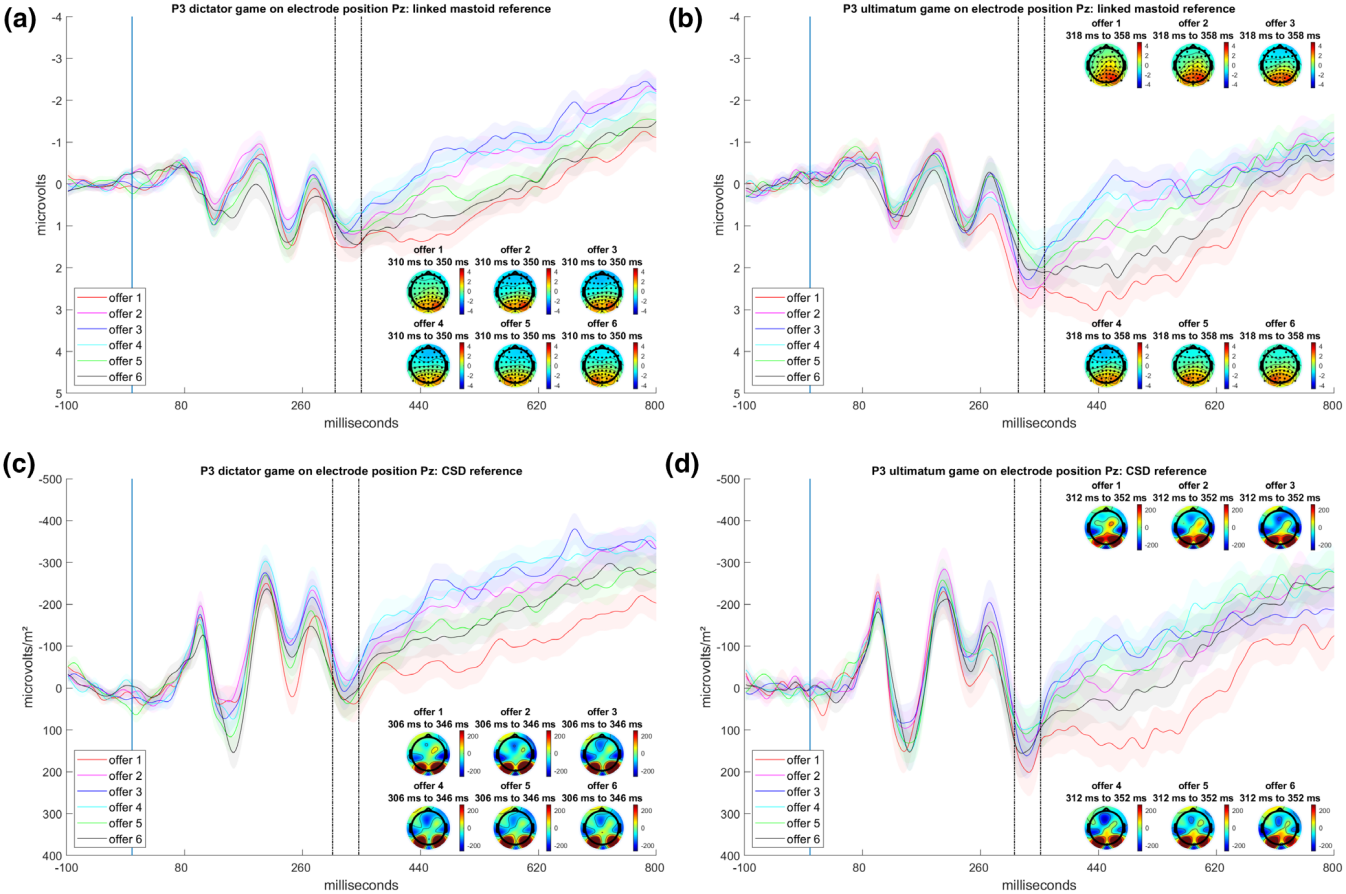
(a) P3 response in the dictator game (DG) with linked mastoid reference. (b) P3 response in the DG with CSD reference. (c) P3 response in the ultimatum game (UG) with linked mastoid reference. (d) P3 response in the UG with CSD reference. Shaded error bars indicate the between SEM.

Post hoc tests in the extension_time‐window_ analysis indicated the highest P3 amplitude for the lowest offer (*p*s < .001), followed by the second‐lowest and the highest offer (*p*s < .01), which again did not differ (*p* = .123), and then the five‐ and three‐credit offers (*p*s < .05), which also did not differ from each other (*p* = .890). The four‐credit offer showed the lowest P3 (*p*s < .05). A graphical illustration is found in Figure 5a,b.

In the extension_CSD_ analysis, post hoc tests indicated that the one‐credit and two‐credit offers led to higher P3 amplitudes than the four‐credit or higher offers (*p*s < .01), except for the two‐credit offer, which did not differ from the five‐credit offer (*p* = .079). A graphical illustration is found in Figure 5c,d.

**FIGURE 6:**
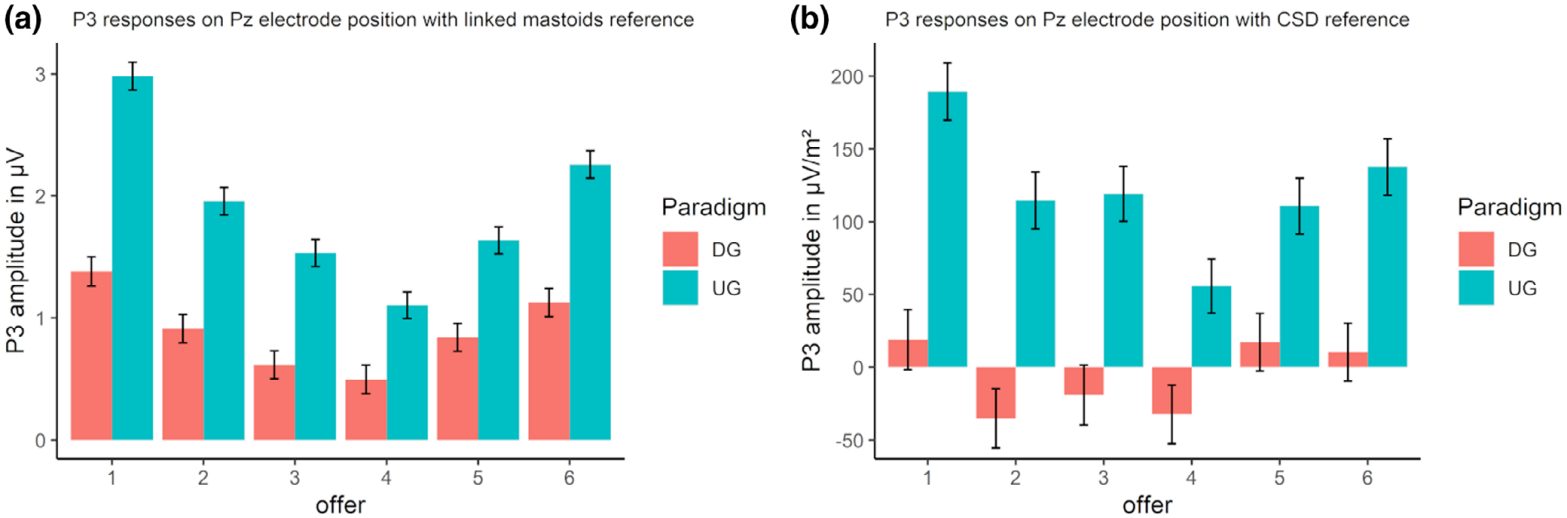
(a) P3 amplitude for offers in the ultimatum game (UG) and the dictator game (DG) with linked mastoids reference. (b) P3 response for offers in the UG and the DG with CSD reference on FCz. Error bars depict the mean within SEM.

In the single‐trial analyses, the best‐fitting model for the linked mastoid‐referenced and the CSD‐referenced data, both with the new peak‐time window quantification, was the additive model accounting for both the offer and the paradigm (see Table S3). Additionally, a U‐shaped P3 response pattern was detected for the offers (see Figure 6a and Table S4), with the lowest offer leading to the highest P3 amplitude (*p*s < .05). The pattern then systematically degraded to the four‐credit offer, which showed the lowest P3 response, before increasing again for the five‐ and six‐credit offers, leading to higher P3 responses (see Table S4).

Using CSD reference, the pattern was similar: the lowest offer led to the highest P3 (*p*s < .05), except for the two highest offers (*p*s > .288). Then, the pattern systematically degraded to the four‐credit offer, which showed the lowest P3 response (*p*s < .05). However, the P3 response to the four‐credit offer did not significantly differ from those to the five‐, three, and two‐credit offers (see Figure 6b and Table S4).

In summary, the offer effects for P3 are in line with target (c) of the replication, indicating higher P3 responses to both the most unfair and most fair offers.

##### Paradigm

In the replication (*F*(1,104) = 8.17, *p* < .01, $\eta_{p}^{2}$ = 0.07), the extension_time‐window_ (*F*(1,104) = 8.80, *p* < .01, $\eta_{p}^{2}$ = 0.08), and the extension_CSD_ analyses (*F*(1,104) = 4.57, *p* < .05, $\eta_{p}^{2}$ = 0.04), the P3 responses were higher in the UG compared with the DG.

In the single‐trial analysis with linked mastoid reference and CSD, the selected model revealed a significant difference for the UG and DG, with higher P3 responses in the UG (Figure 6).

The details concerning anteriority, laterality, and their interactions with offer and paradigm are mentioned in the Supplemental Materials S11.

### 3.3 Valence and arousal ratings

The ANOVA for the valence ratings revealed a main effect for the fairness of the offers (*F*(5,520) = 285.48, *p* < .001, $\eta_{p}^{2}$ = 0.73, GG = 0.40), with higher offers leading to higher valence (*p*s < .001). No other effects were significant (*F*s ≤ 1.84).

**FIGURE 7:**
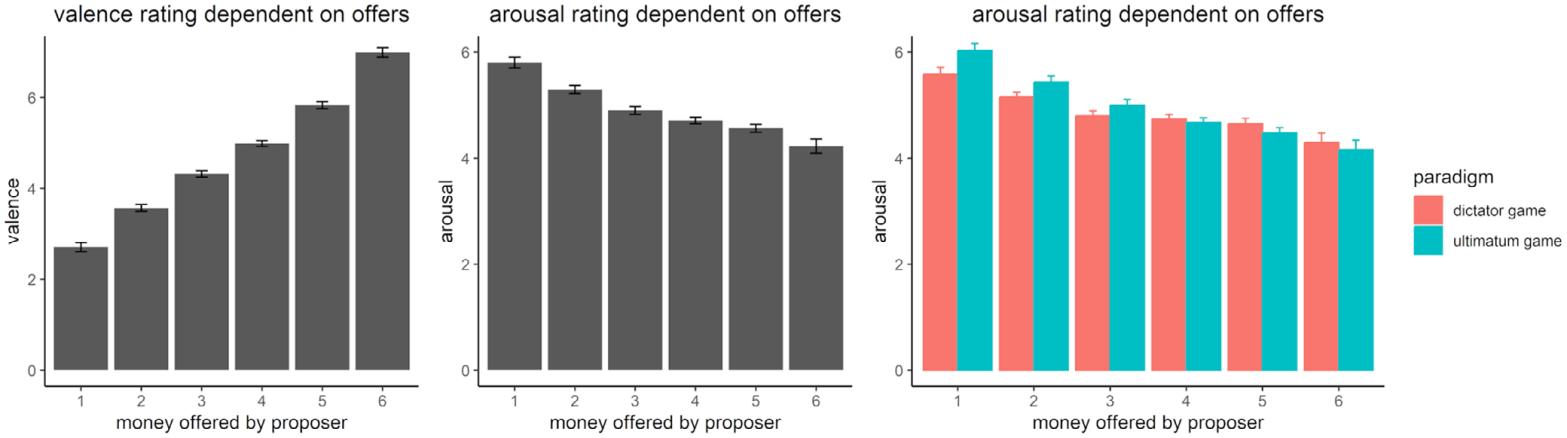
Subjective ratings dependent on the paradigm and fairness of the offer. Error bars depict the mean within SEM.

In the ANOVA for the arousal ratings, the main effect of the fairness of the offers was also significant (*F*(5,520) = 32.19, *p* < .001, $\eta_{p}^{2}$ = 0.24, GG = 0.36; see Figure 7), revealing a gradient with the highest arousal toward the worst offers (*p*s < .05). However, there were no significant differences between the 3:9, 4:8, and 5:7 offers (*p*s > .091), nor between the 4:8 and 5:7 offers (*p* = .398) or the 5:7 and 6:6 offers (*p* = .091). The significant interaction between the fairness of the offer and the paradigm (*F*(5,520) = 4.49, *p* < .001, $\eta_{p}^{2}$ = 0.04, GG = 0.77) revealed that for the UG and DG, the lowest offer elicited higher arousal than all other offers (*p*s < .01) except for the second‐worst offer (*ps* ≥ .181). For the UG, the second‐lowest offer (2:10) differed from all offers >three credits (*p*s < .05). Additionally, the 3:9 offer was different from the 6:6 offer (*p* = .003). In the DG, apart from the lowest offer, only the second‐lowest (2:10) differed from the 6:6 offer (*p* = .01). Taken together, these findings are in line with target (d) of the replication, which expected that more negative emotional ratings predict higher rejection rates.

### 3.4 SCR results

**FIGURE 8:**
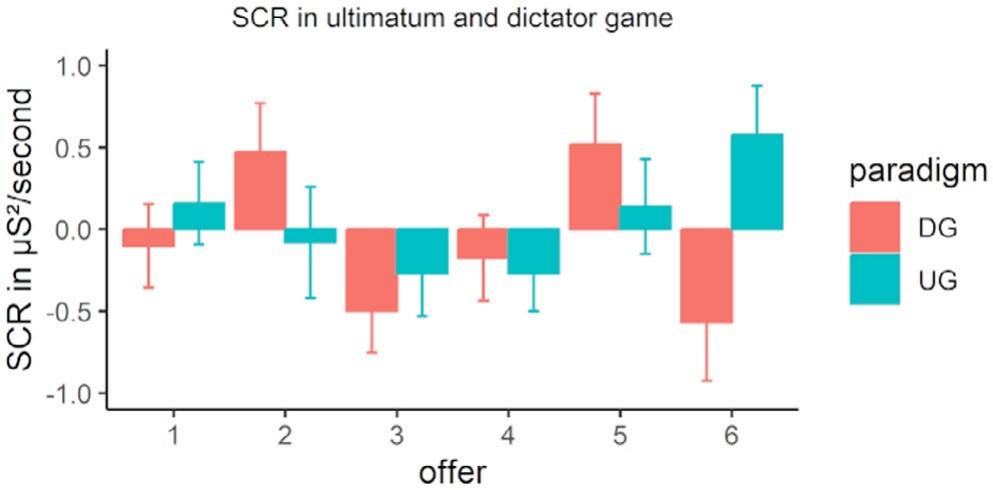
Skin conductance response (SCR) response in the dictator game (DG) and the ultimatum game (UG).

The SCR results revealed no significant main effect of offer (*F*(5,250) = 1.60, *p* = .158, $\eta_{p}^{2}$ = 0.02) and paradigm (*F*(1,104) = 1.50, *p* = .224, $\eta_{p}^{2}$ = 0.01), but an interaction between offer and paradigm (*F*(5,250) = 2.56, *p* = .027, $\eta_{p}^{2}$ = 0.02, GG = 0.78; see Figure 8). Although there was no effect of the offer in the UG (*F*(5,250) = 1.35, *p* = .24, $\eta_{p}^{2}$ = 0.01), offer had an effect in the DG (*F*(5,250) = 2.70, *p* = 0.020, $\eta_{p}^{2}$ = 0.03). However, post hoc *t* tests did not show a significant effect pattern. Taken together, these findings are not in line with target (e) of the replication, which stated that larger SCRs would predict higher rejection rates.

Exploring the reliability using the ICC revealed that the SCR showed poor reliability in every offer condition (see Table 3). This was partly expected due to the acclimatization to the conditions. Yet, the magnitude of this effect was much higher than initially assumed.

**TABLE 3:** Test–retest reliability (ICC) of SCR responses to different offers in the two paradigms.

Paradigm	Offer	Intraclass correlation	Lower bound 95% CI	Upper bound 95% CI
UG	1	.00	−.24	.22
	2	.00	−.24	.22
	3	.32	.16	.47
	4	.00	−.24	.22
	5	.00	−.24	.22
	6	.00	−.24	.22
DG	1	.00	−.24	.22
	2	.00	−.24	.22
	3	.00	−.24	.22
	4	.39	.24	.52
	5	.00	−.24	.22
	6	.00	−.23	.21

Abbreviations: DG, dictator game; ICC, intraclass correlation coefficient; UG, ultimatum game.

### 3.5 Multiple regression

The stepwise linear regression revealed that only the valence rating contributed significantly to the variance explained by the MLM (see model 1 in Table 4).

**TABLE 4:** Results of the stepwise linear regression models.

Model fit measures			Comparison to model 1				
Model	*R*	*R* ^2^	Δ*R* ^2^	*F*	df1	df2	*p*
1	.37	.14					
2	.41	.17	.03	3.87	1	102	.052
3	.42	.18	.01	0.77	1	101	.383

*Note*: Model 1 variables: (constant) and valence ratings. Model 2 variables: (constant), valence ratings, and skin conductance responses. Model 3 variables: (constant), valence ratings, skin conductance responses, and feedback‐related negativity at electrode FCz for 6:6 in the ultimatum game. Dependent variable: rejections at 10:2 offers.

The valence rating significantly predicted the acceptance behavior (*t*(104) = 4.06, *p* < .001, *β* = .37). This is not in line with target (f) of the replication, which expected that taken together, FRN amplitudes to fair offers, SCR amplitude, and emotional valence ratings would account for ≥50% of variance in individual differences in rejection rates for the 10:2 offer.

### 3.6 Single‐trial midfrontal theta activity

**FIGURE 9:**
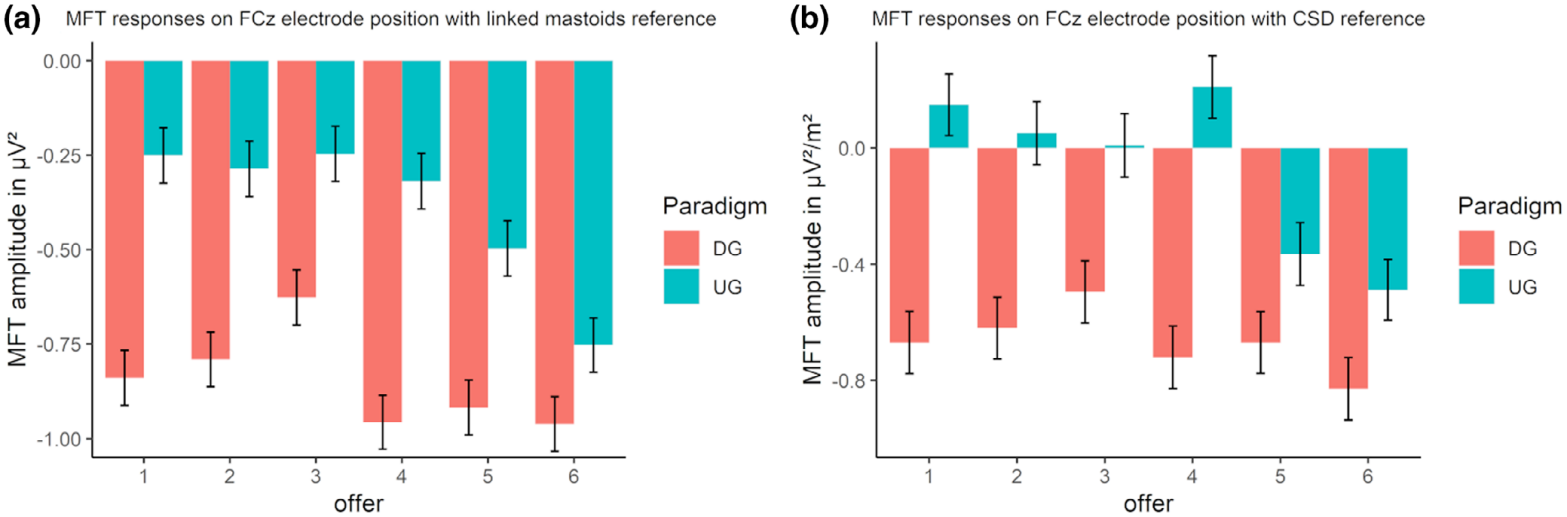
(a) Midfrontal theta (MFT) response for offers in ultimatum game (UG) and dictator game (DG) with linked mastoids reference on FCz. (b) The same twofold interaction with CSD reference on FCz. Error bars depict mean within SEM.

**FIGURE 10:**
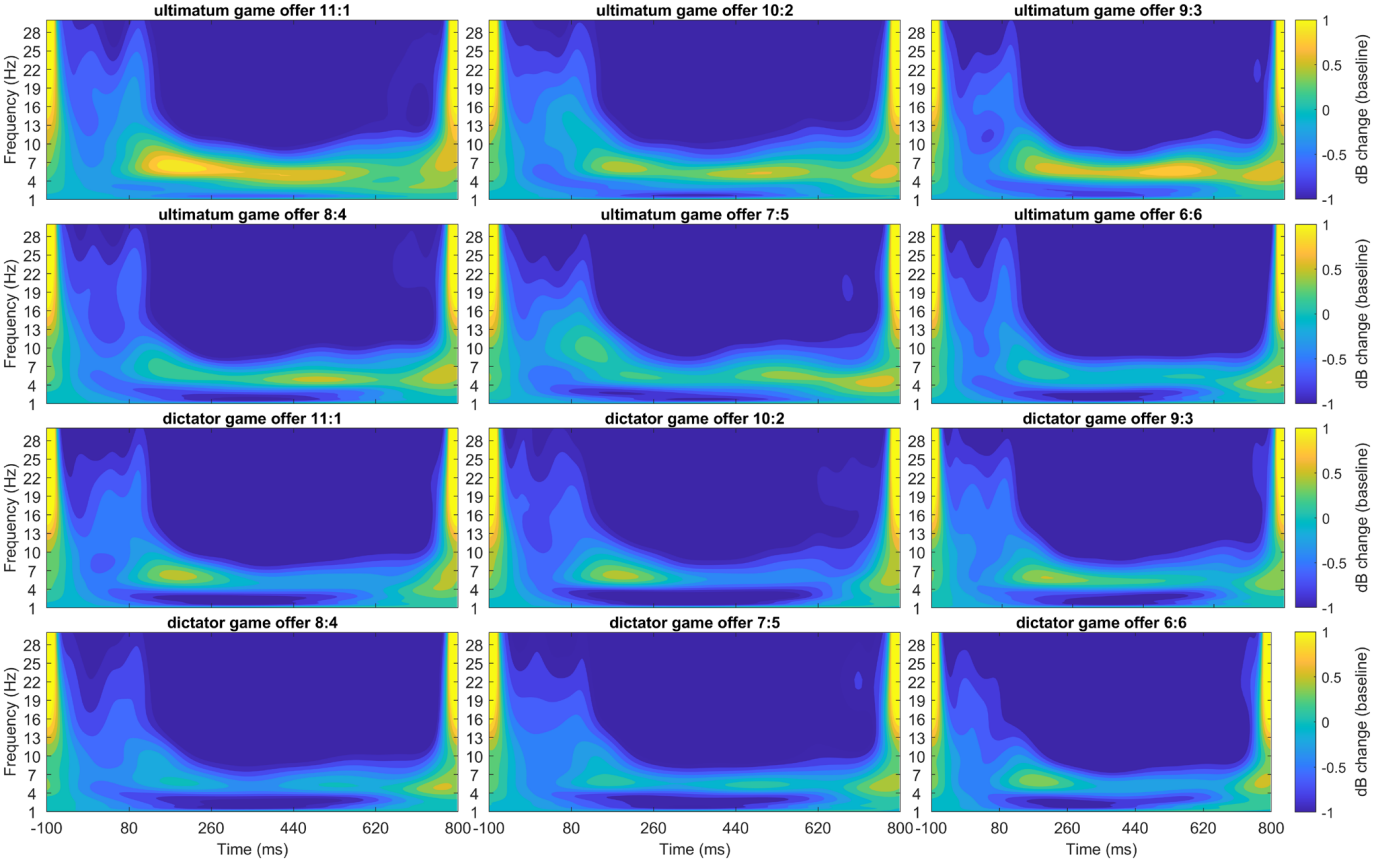
Midfrontal theta (MFT) time frequency response for offers in ultimatum game and dictator game with linked mastoid reference on FCz.

The best‐fitting model for the linked mastoid‐referenced data was the additive model accounting for both the offer and the paradigm (see Figures 9a and 10, and Table S5). The model revealed a significant difference between the UG and the DG, with higher MFT responses in the UG. Additionally, the MFT reaction for the six‐credit offer was lower than for the three‐ and four‐credit offers (*p*s < .05, see Table S6).

**FIGURE 11:**
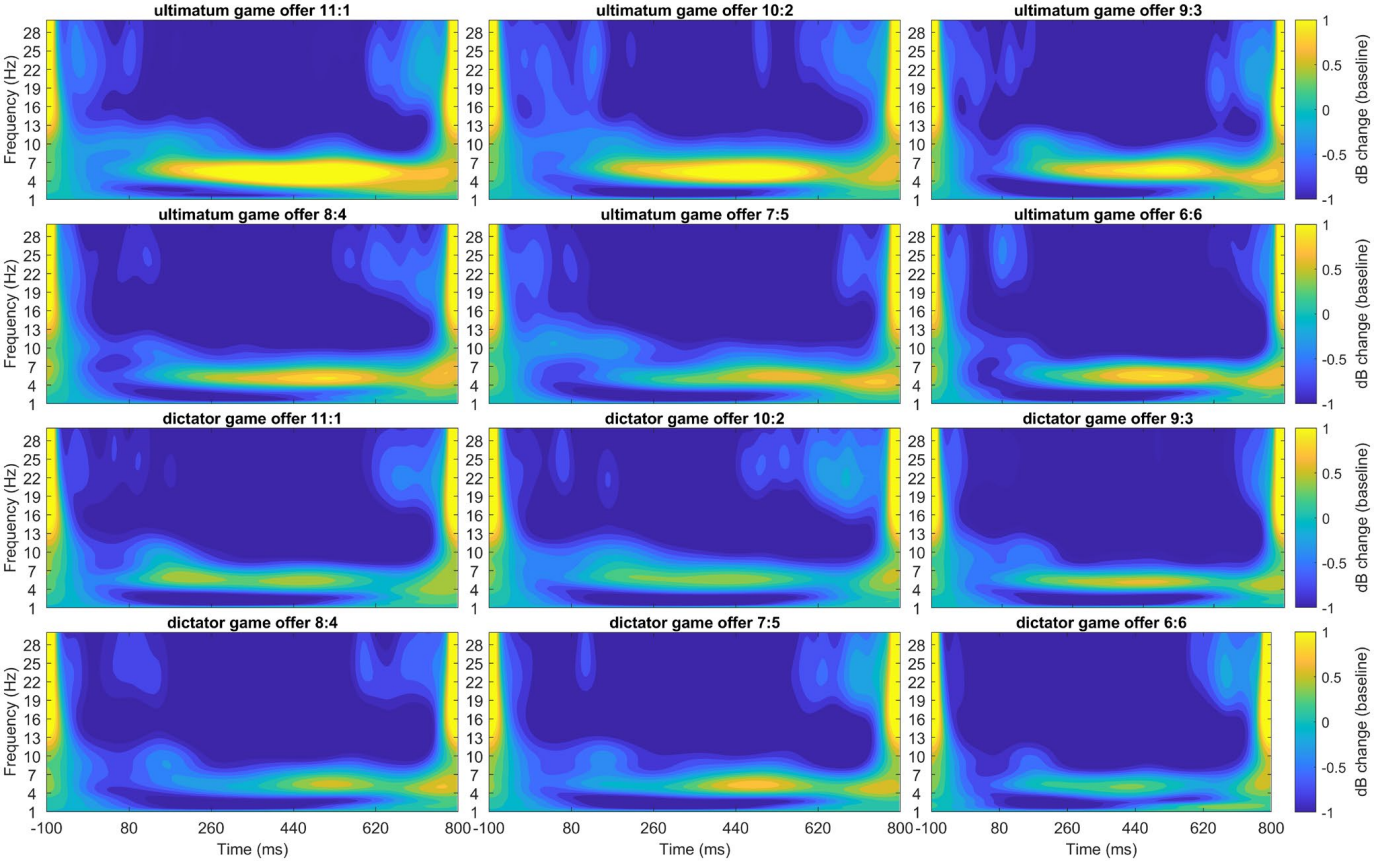
Midfrontal theta (MFT) time frequency response for offers in ultimatum game and dictator game with CSD reference on FCz.

The best‐fitting model for the CSD‐referenced data was the interaction model accounting for the offer and the paradigm (see Figures 9b and 11, and Table S5). The model revealed a significant difference for the UG and DG, with higher MFT responses in the UG. Additionally, the MFT reaction for the six‐credit offer was lower than for all other offers (*p*s < .01), except for the five‐credit offer (*p* = .739, see Table S6). The interaction between paradigm and offer revealed that in the DG, the offers did not differ in MFT activation. In the UG, however, the five‐ and six‐credit offers produced lower MFT responses than all other offers (*p*s < .05), except for a lack of difference between the three‐ and five‐credit offers (*p* = .267). A comprehensive display of all effects in the best‐fitting models is found in Table S7.

### 3.7 Prediction of behavior single‐trial analysis

The best‐fitting model for the linked mastoid‐referenced data was the interaction model including the midfrontal theta activation per trial, mean midfrontal theta per participant, and the offer as predictor (see Table S8).

The higher the offer, the higher the chance of acceptance (*p*s < .001). The average MFT per participant was associated with a lower acceptance in general (*p* < .001), which was systematically attenuated for more fair offers (*p*s < .001) and high MFT reactions per trial (*p* < .01). Accordingly, the influence of mean MFT per participant was weaker in fairer trials and in trials with high MFT. There was an additional interaction between MFT per trial and the highest offer (*p* < .01), leading to less acceptance with higher MFT, particularly if high MFT per participant was also present (*p* < .05). Finally, rejection was also more pronounced toward the second‐lowest offer if both MFT per participant and MFT per trial were high.

For the CSD‐referenced data, too, the best‐fitting model was the interaction model including the midfrontal theta activation per trial, mean midfrontal theta per participant, and the offer as predictor (see Table S8).

**FIGURE 12:**
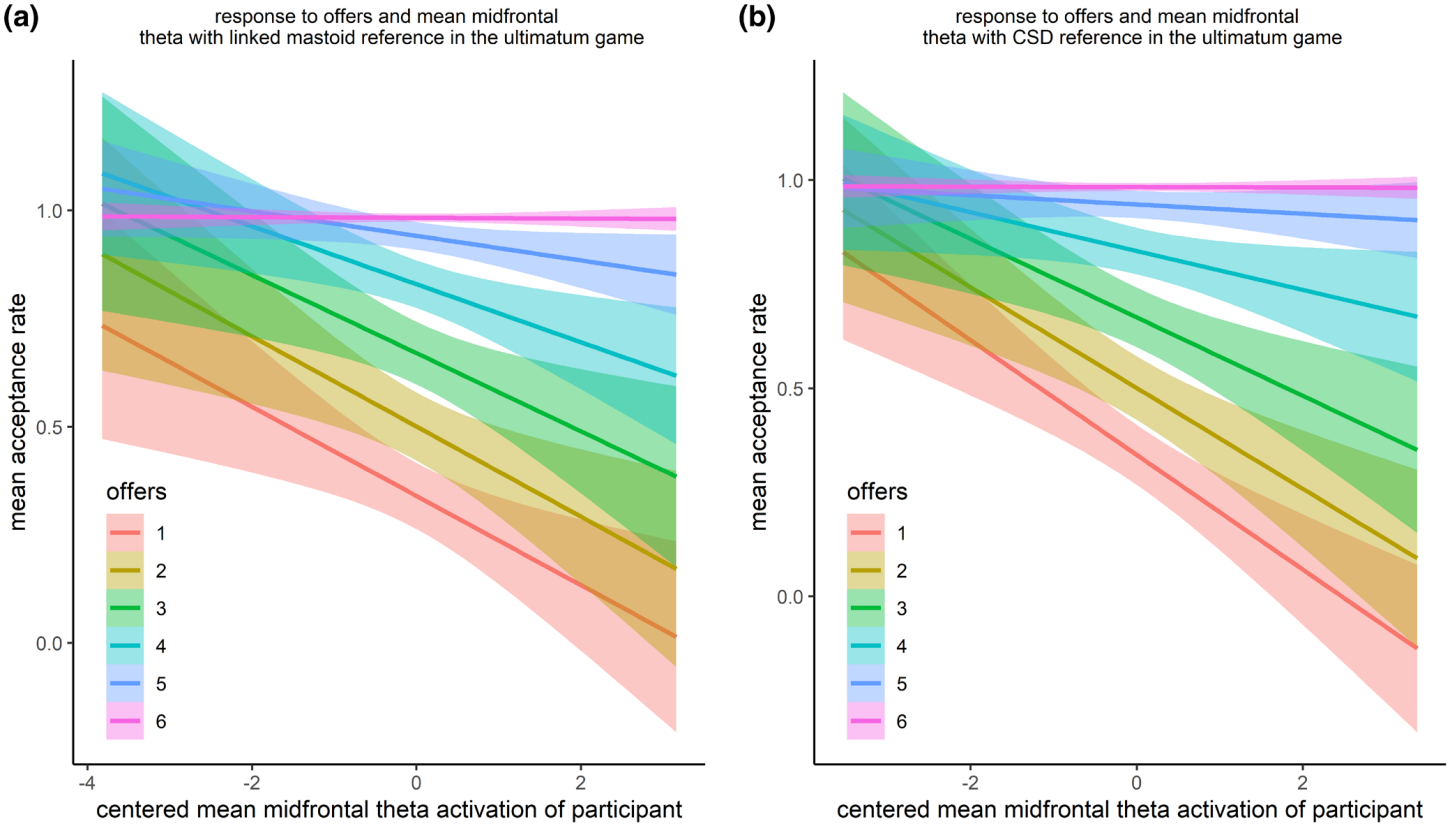
Mean acceptance rate depending on offers and mean midfrontal theta activation for linked mastoid reference (a) and CSD reference (b). Shaded error bars display mean within SEM.

The higher the offer, the higher the chance of acceptance (*p*s < .001). The mean MFT per participant led to a lower acceptance in general (*p* < .001). This influence was systematically attenuated for more fair offers (*p*s < .001). Accordingly, the influence of mean MFT per participant was weaker in fairer trials and in trials with high MFT. For the MFT reaction per trial, there was no main effect. However, there was an additional interaction with the highest offer (*p* < .01), leading to less acceptance with higher MFT. A graphical illustration of the effects above is presented in Figure 12. A complete display of all effects in the best models is found in Table S9.

For the exploratory addition of the acceptance button position and the valence ratings to the model, the best‐fitting models for mastoid and CSD reference included both exploratory variables (see Table S8). To account for the complexity of the model (194 terms), only Bonferroni‐adjusted significant effects are interpreted, but a complete display of the model terms is found in Table S10. In addition to the previously detected offer effects, the mean valence rating per participant indicated more acceptance for higher valence ratings in general (*p*s < .001) in both reference schemes. It even amplified if the valence rating was high in this type of trial for CSD reference (*p* < .001). Concerning the general effect of mean MFT per participant leading to lower acceptance, this effect was preserved for CSD reference (*p* < .001) but not for linked mastoids (*p* = .061). The previously observed attenuating influence of the offers on this effect was not evident (*p*s > .006). However, an amplification of the rejection dependent on the offer was identified if the mean valence rating per participant was high (*p*s < .001), both for CSD and for linked mastoids for the three‐, four‐, and five‐credit offers (*p*s < .001). This effect was particularly strong for the three‐credit offer and if the valence rating of this trial was high (*p*s < .001). The attenuating effects of rejection‐related mean MFT per participant were now observed if both a high mean valence per participant and a high mean MFT per participant were given (*p*s < .001). Furthermore, the effect was evident when high valence ratings were reported in the respective trial type within this combination (*p*s < .001).

Concerning the influence of the position of the acceptance button, no main effect or simple twofold interaction but a threefold interaction for the linked mastoid reference were observed, revealing a link between mean MFT per participant and rejection in the two‐ and three‐credit offers, but only if the accept button was on the right side (*p*s < .001). This effect was further amplified for the two‐credit offer if the valence rating was high, both in the respective type of trial and per participant in general (*p* < .001). Yet, for the three‐credit offer, this position of the button in combination with the valence ratings led to the opposite effect, hence more acceptance (*p* < .001) for CSD, and exploratorily, for linked mastoids (*p* = .007).

The explained variance of all models is seen in Table S8. Remarkably, the final model including the valence ratings and the acceptance button explains the data rather well, with *R* ^2^_conditional_ = .93 for CSD and *R* ^2^_conditional_ = .93 for linked mastoid reference as well as *R* ^2^_marginal_ = .66 for CSD and *R* ^2^_marginal_ = .58 for linked mastoid reference (cf. Nakagawa & Schielzeth, 2013). The *R* ^2^_conditional_ and *R* ^2^_marginal_ of all models are seen in Table S8.

## 4 DISCUSSION

Replication is essential for scientific progress, as it contributes to the development of more robust evidence and thus to the establishment of a more solid foundation on which to build future research. In this study, we replicated the seminal experiment conducted by Hewig et al. (2011), in which participants played both the ultimatum and DG. The primary objective was to uncover the affective correlates of economic decisions. Although we could show psychophysiological correlates of why humans deviate from rational choice in line with the original study, there were substantial differences in the effect patterns. Our findings replicated that unfair offers in the one‐shot UG were rejected more often and elicited more negative subjective emotional ratings (targets a and d). However, in contrast to Hewig et al. (2011), there were no meaningful effects on the SCRs, neither in the UG nor in the DG (i.e., target e failed). Additionally, our data supports a quadratic effect for the offers on the FRN and the P3. Although this was also the case in the original study for P3 (target c), the pattern was different for FRN (i.e., target b failed). Furthermore, multiple regression did not explain the expected amount of variance (i.e., target f failed). Taken together, only half of the six targets were successfully replicated. Expanding the original study, midfrontal theta activity showed a linear increase with increasing fairness of the offer and successfully predicted acceptance behavior in the UG.

Importantly, we were able to replicate the key results of behavior and emotional ratings (i.e., valence and arousal) reported by Hewig et al. (2011). With increasing fairness of the offer in the UG, acceptance rates and valence ratings increased. As an extension, we also demonstrated that arousal ratings decreased with increasing fairness of the offer. In contrast to the original study, however, the stepwise regression of valence and psychophysiological correlates (i.e., SCRs and FRN) of this replication study did not confirm the substantial independent amount of variance explained by each predictor in the 10:2 offers in the UG. Here, only the model including the valence rating as a single predictor was significant. The lack of the effect of FRN and SCR may be traced back to different factors. For example, the small sample size of the original study might have biased the true effects of the prediction models. Additionally, the measurements of SCR taken from the foot (original study) versus the hand (replication) may differ fundamentally. In early studies of SCR, it was suggested that foot measurement might offer advantages in terms of amplitude (Edwards, 1988), yet more recent research challenges these results, particularly regarding the distal phalange measurement used in our study (Payne et al., 2013). Still, the lack of variance explanation in our results may be attributed to differences in electrode placement compared with the original study. Moreover, SCRs showed no meaningful effect pattern in the current study. Although the results reported by Hewig et al. (2011) have already been conceptually replicated in a larger sample compared with the original (*N* = 54; T. Wu et al., 2013), other findings indicate that individual differences may also play a central role (e.g., interoception; see Dunn et al., 2012). This could be an additional factor influencing the present results, given that the present sample appears to be more diverse than in the original study.

The largest deviation from the original study emerged in the analyses of electrocortical correlates of decision‐making. The proposed linear relation between the offer and the FRN response—which has also been documented in more recent research (e.g., Rodrigues et al., 2022; Weiß, Rodrigues, et al., 2020b)—was not found in the current replication. In contrast, a quadratic relation of the FRN response emerged (e.g., see also Mussel et al., 2014; Weiß, Mussel, & Hewig, 2020a), challenging the binary evaluation of positive versus negative outcomes proposed in economic games (Hajcak et al., 2006). Especially in the UG, strategic decisions change the perception (P2 component) and evaluation process from offer fairness to anticipatory satisfaction of punishment (Mussel et al., 2022). The interaction between paradigm, offer, and anteriority as reported for the extension_time‐window_ showed that the effect of less negative FRN responses for low offers were only present in the UG and not in the DG. This finding supports the idea that in the UG, the anticipation of the subsequent successful punishment that will be performed by the participant may already be foreshadowing FRN responses to low offers. However, on an offer‐independent level, despite having increased the DG trials to allow for robust comparability between the two economic games, we did not observe a significant main effect between the UG and DG. The absence of substantial differences in FRN between the UG and DG are in line with other research (see Rodrigues et al., 2022; Zhong et al., 2019) and might indicate that the FRN simply captures a general evaluation of the offer, irrespective of the opportunity to act or even punish the interaction partner for their unfair behavior (cf. Mussel et al., 2022). Yet, the quadratic relationship concerning the FRN response may simply code the anticipation of successful punishment, driven by the low offers in the UG paradigm in our study.

With regard to the P3 component, we could replicate the U‐shaped neural response pattern for the offers reported in Hewig et al. (2011). Thus, the pattern for FRN and P3 responses toward the offer presentation was comparable in the original and current study. In contrast to previous research (Zhong et al., 2019), our findings indicated that the UG elicits larger P3 amplitudes than the DG. As the P3 brain potential is related to the allocation of attentional resources in the decision‐making process (e.g., Falco et al., 2019), the observed difference between the paradigms might reflect a higher relevance of UG offers compared with DG offers for behavioral responses. This is in line with research positing the P3 as a marker of motivational states (e.g., Nieuwenhuis et al., 2005), given that the task of deciding between accepting or rejecting an offer in the UG holds greater motivational relevance compared with the passive act of doing nothing (except, perhaps, for a trivial button press) in the DG.

FRN and P3 responses showed several interactions between offer or paradigm with the anterior and lateral factors. For P3, the highest amplitudes and offer‐dependent patterns were found at posterior sites, with a tendency to shift to the right sites for linked mastoids, but similar patterns on electrode position CPz for CSD reference (see Supplemental Materials S11: Figure S11c). This concentration to the midline in CSD could be expected, as the CSD acts as a spatial filter amplifying the differentiation between activation patterns (cf. Cohen, 2014; Kayser, 2009). What was unexpected, however, was the shift to the slightly more central position, emphasizing the importance of comparing references. Additionally, the same effect pattern was found on Pz, albeit with different amplitudes (see Supplemental Materials S11: Figure S11c). Yet, the CSD reference picked up different variance patterns concerning differences of P3 amplitudes between the two paradigms on the midline electrode sites (see Supplemental Materials S11: Figure S11b), preserving the pattern found for linked mastoids on the posterior electrodes while stressing the importance of recognizing topographical representation in different reference schemes (cf. Figures 3 and 5). For the FRN, similar results were observed concerning the pattern's dependency on the reference. The most negative and offer‐dependent patterns were identified at all frontal and frontocentral sites for linked mastoid reference, whereas similar patterns were most pronounced at the central, frontal, and frontocentral electrodes for CSD (cf. Supplemental Materials S11: Figure S11a). This again emphasizes the importance of controlling for the topographical distributions of effect patterns dependent on reference schemes. Importantly, the “classical” topographical target electrodes (FRN: Fz/FCz, P3: Pz) yielded similar effect patterns for both references, which is reassuring in terms of both reliability and replicability.

As an extension to the original study, we analyzed MFT activity. Similar to the P3, MFT was significantly larger in the UG compared with the DG. While the FRN mirrors the evaluation of the offers, MFT reflects the cognitive control component in the decision process (Cavanagh & Frank, 2014; Cohen, 2011). In more detail, the cognitive control required to overcome the default behavior of accepting the offer in the UG (vs. DG) may have resulted in higher MFT responses (Rodrigues et al., 2022). As hypothesized, MFT—in interaction with offer—served as a predictor for behavioral responses in the UG. The lower the offer and the higher the MFT activity, the lower the acceptance behavior. This aligns with research showing that MFT is associated with adjustment of the subsequent action (van de Vijver et al., 2011), indicating an absence of cognitive control behavior for low monetary offers (i.e., no adaption during the game to maximize the outcome; Billeke et al., 2014). In the present study, we exploratorily modulated this phenomenon with valence ratings. High valence ratings, either in general or in specific trials, contributed to more acceptance, whereas higher valence ratings in general may also have indicated a higher expectation of getting good offers. Accordingly, the observed effect of higher MFT leading to lower acceptance rates was further amplified in these individuals, particularly for the low offers, and for both references. Hence, the valence ratings also contributed to the explained variance and helped in identifying specific circumstances that further enhance the revealed patterns of offer and MFT‐dependent rejection behavior. A surprising finding in the present data is that the relevance of MFT responses for rejection is not determined by trial‐based MFT responses but by participant‐dependent mean MFT responses. This is in line with the importance of these trait‐like (or prolonged‐state‐like) mean MFT responses for punishment behavior in other paradigms such as the third‐party DG (Rodrigues et al., 2020). The cognitive control depicted by the mean MFT may lead participants to choose not to follow their initial impulses and default behavior (in the case of our study, accepting the offer) but to override this response in favor of rejection.

Lastly, we would like to highlight several limitations of the current study. Firstly, we would have preferred a different presentation style of the offers, that is, pie charts instead of divided color bars. Pie charts are presented as a focal object and are thus potentially easier to understand. Secondly, we tested more women compared with men, which limits the generalizability of the results. However, the gender ratio was comparable to the original study (original: 66.66% female, replication: 63.81% female). Finally, we cannot rule out that the tone presented in the original study might have affected the results, potentially leading to deviations in physiological responses in this replication, especially regarding the SCR results. Still, we decided against incorporating this additional stimulus as our goal was to investigate the affective and psychophysiological correlates of economic decision‐making in the most unbiased way possible.

In summary, we conducted a replication and extension of Hewig et al.'s (2011) experiment on the affective components of economic decision‐making. Although the general behavioral and subjectively rated patterns where comparable, the neural results differed to some extent. We conclude that the replication was successful in the sense that we could conceptually replicate the finding that human behavior is not rational, yet unfolds in a somewhat different way from what Hewig et al. (2011) had originally proposed.

## AUTHOR CONTRIBUTIONS

**Johannes Rodrigues:** Conceptualization; data curation; formal analysis; funding acquisition; investigation; methodology; project administration; validation; visualization; writing – original draft; writing – review and editing. **Martin Weiß:** Conceptualization; writing – original draft; writing – review and editing. **Grit Hein:** Supervision; writing – review and editing. **Johannes Hewig:** Conceptualization; funding acquisition; resources; software; supervision; writing – review and editing.

## FUNDING INFORMATION

This article was funded by Julius‐Maximilians‐Universität Würzburg (Faculty of human sciences: Research funding) and the European Union through the project “Hyperautomation Ökosystem (Hyko)” (1.2‐StMWKF.4‐UFR‐002) of the European Regional Development Fund (ERDF).

## CONFLICT OF INTEREST STATEMENT

None.

## PRE‐REGISTER

https://doi.org/10.17605/OSF.IO/VK93G.

## Supporting information


**Table S1.** Fit indices (AICc) and p‐information loss for the FRN component.
**Table S2.** Effects on offer for the FRN.
**Table S3.** Fit indices (AICc) and p‐information loss for the P3 component.
**Table S4.** Effects on offer for the P3.
**Table S5.** Fit indices (AICc) and p‐information loss for the midfrontal theta band response.
**Table S6.** Effects on offer for the midfrontal theta band response.
**Table S7.** Effects for best fitting model concerning midfrontal theta responses.
**Table S8.** Fit indices (AICc) and p‐information loss for the behavioral response.
**Table S9.** Effects for best fitting model concerning behavioral responses.
**Table S10.** Effects for best fitting model concerning behavioral responses with acceptance button positions.
**Supplemental Materials S11.** Detailed topographical results for FRN and P3.
**Figure S11a.** A. Threefold interaction between, offer, anteriority, and laterality for FRN signal. B. The same threefold interaction with the new quantification CSD reference. Error‐bars depict mean within SEM.
**FigureS11b.** A. P3 threefold interaction between paradigm, anteriority, and laterality using the new time window quantification with linked mastoid reference. B. The same interaction using the new time window quantification with CSD reference. Error‐bars depict the mean.
**Figure S11c.** A. P3 threefold interaction offer, anteriority, and laterality, using the new time window quantification with linked mastoid reference. B. The same interaction using the new time window quantification with CSD reference. Error‐bars depict the mean within SEM.

## Data Availability

The data and scripts can be found at https://osf.io/zq5ej/.
