# Supplementary material for: Electrophysiological correlates of why humans deviate from rational decision‐making: A registered replication study
Source: Psychophysiology. 2024 Aug 13;62(1):e14665. doi: 10.1111/psyp.14665 (PMC11775885; doi:10.1111/psyp.14665)
Supplement: Supplementary file 1 — Table S1. Fit indices (AICc) and p‐information loss for the FRN component. Table S2. Effects on offer for the FRN. Table S3. Fit indices (AICc) and p‐information loss for the P3 component. Table S4. Effects on offer for the P3. Table S5. Fit indices (AICc) and p‐information loss for the midfrontal theta band response. Table S6. Effects on offer for the midfrontal theta band response. Table S7. Effects for best fitting model concerning midfrontal theta responses. Table S8. Fit indices (AICc) and p‐information loss for the behavioral response. Table S9. Effects for best fitting model concerning behavioral responses. Table S10. Effects for best fitting model concerning behavioral responses with acceptance button positions. Supplemental Materials S11. Detailed topographical results for FRN and P3. Figure S11a. A. Threefold interaction between, offer, anteriority, and laterality for FRN signal. B. The same threefold interaction with the new quantification CSD reference. Error‐bars depict mean within SEM. FigureS11b. A. P3 threefold interaction between paradigm, anteriority, and laterality using the new time window quantification with linked mastoid reference. B. The same interaction using the new time window quantification with CSD reference. Error‐bars depict the mean. Figure S11c. A. P3 threefold interaction offer, anteriority, and laterality, using the new time window quantification with linked mastoid reference. B. The same interaction using the new time window quantification with CSD reference. Error‐bars depict the mean within SEM. [file PSYP-62-e14665-s001.docx]

**Supplemental Materials:**

**Electrophysiological correlates of why humans deviate from rational decision making: A registered replication study**

**Content:**

- Table S1: Fit indices (AICc) and p-information loss for the FRN component. *(Page S2)*
- Table S2: Effects on offer for the FRN. *(Page S2)*
- Table S3: Fit indices (AICc) and p-information loss for the P3 component. *(Page S2)*
- Table S4: Effects on offer for the P3. *(Page S3)*
- Table S5: Fit indices (AICc) and p-information loss for the midfrontal theta band response. *(Page S3)*
- Table S6: Effects on offer for the midfrontal theta band response. *(Page S4)*
- Table S7: Effects for best fitting model concerning midfrontal theta responses. *(Page S5)*
- Table S8: Fit indices (AICc) and p-information loss for the behavioral response. *(Page S6)*
- Table S9: Effects for best fitting model concerning behavioral responses. *(Page S7)*
- Table S10: Effects for best fitting model concerning behavioral responses with acceptance button positions. *(Pages S8 - S14)*
- Supplemental materials S11: Detailed topographical results for FRN and P3. *(Pages S15 -24)*
  - Figure S11a: A. Threefold interaction between, offer, anteriority, and laterality for FRN signal. B. The same threefold interaction with the new quantification CSD reference. Error-bars depict mean within SEM.
  - FigureS11b: A. P3 threefold interaction between paradigm, anteriority, and laterality using the new time window quantification with linked mastoid reference. B. The same interaction using the new time window quantification with CSD reference. Error-bars depict the mean.
  - Figure S11c: A. P3 threefold interaction offer, anteriority, and laterality, using the new time window quantification with linked mastoid reference. B. The same interaction using the new time window quantification with CSD reference. Error-bars depict the mean within SEM.

Table S1: Fit indices (AICc) and p-information loss for the FRN component.

| **random effect** | **fixed effect** | **AIC  CSD reference** | **AIC  mastoid reference** | **Probability of no information loss to best model overall CSD reference** | **Probability of no information loss to best model overall mastoid reference** |
| --- | --- | --- | --- | --- | --- |
| 1\|per subject/paradigm |  | 111384.4 | 280006.9 | 0.000 | 0.000 |
| 1\|per subject/paradigm | offer | 111346.7 | 279922.6 | 0.067 | 1.000 |
| 1\|per subject/paradigm | paradigm | 111385.8 | 280008.9 | 0.000 | 0.000 |
| 1\|per subject/paradigm | paradigm+offer | 111348 | 279924.6 | 0.035 | 0.368 |
| 1\|per subject/paradigm | paradigm*offer | 111341.3 | 279922.6 | 1.000 | 1.000 |

*Note: Green marks the best and simplest model. Orange marks the best and simplest model that is not sufficiently different from the best complex model (see p-value information loss).*

Table S2:Effects on offer for the FRN.

|  | ***linked mastoid reference*** | | | | ***CSD reference*** | | | |
| --- | --- | --- | --- | --- | --- | --- | --- | --- |
| **offer differences** | **Estimate** | **Std. Error** | **z-value** | ***p*-value** | **Estimate** | **Std. Error** | **z-value** | ***p*-value** |
| 2-1 | -1.00 | 0.18 | -5.42 | < 0.001 | -0.08 | 0.02 | -4.41 | < 0.001 |
| 3-1 | -1.23 | 0.18 | -6.67 | < 0.001 | -0.09 | 0.02 | -4.81 | < 0.001 |
| 4-1 | -1.17 | 0.18 | -6.35 | < 0.001 | -0.09 | 0.02 | -4.45 | < 0.001 |
| 5-1 | -0.61 | 0.18 | -3.32 | 0.011 | -0.03 | 0.02 | -1.40 | 0.726 |
| 6-1 | 0.02 | 0.18 | 0.13 | 1 | -0.01 | 0.02 | -0.57 | 0.993 |
| 3-2 | -0.23 | 0.18 | -1.25 | 0.815 | -0.01 | 0.02 | -0.40 | 0.999 |
| 4-2 | -0.17 | 0.18 | -0.92 | 0.942 | 0.00 | 0.02 | -0.04 | 1 |
| 5-2 | 0.39 | 0.18 | 2.11 | 0.284 | 0.06 | 0.02 | 3.01 | 0.031 |
| 6-2 | 1.02 | 0.18 | 5.55 | < 0.001 | 0.07 | 0.02 | 3.84 | 0.002 |
| 4-3 | 0.06 | 0.18 | 0.33 | 0.999 | 0.01 | 0.02 | 0.36 | 0.999 |
| 5-3 | 0.62 | 0.18 | 3.35 | 0.010 | 0.07 | 0.02 | 3.41 | 0.008 |
| 6-3 | 1.25 | 0.18 | 6.80 | < 0.001 | 0.08 | 0.02 | 4.24 | < 0.001 |
| 5-4 | 0.56 | 0.18 | 3.03 | 0.030 | 0.06 | 0.02 | 3.05 | 0.028 |
| 6-4 | 1.19 | 0.18 | 6.48 | < 0.001 | 0.07 | 0.02 | 3.89 | 0.001 |
| 6-5 | 0.63 | 0.18 | 3.45 | 0.007 | 0.02 | 0.02 | 0.84 | 0.961 |

Table S3: Fit indices (AICc) and p-information loss for the P3 component.

| **random effect** | **fixed effect** | **AIC  CSD reference** | **AIC  mastoid reference** | **Probability of no information loss to best model overall CSD reference** | **Probability of no information loss to best model overall mastoid reference** |
| --- | --- | --- | --- | --- | --- |
| 1\|per subject/paradigm |  | 109581.1 | 267209.6 | 0.000 | 0.000 |
| 1\|per subject/paradigm | offer | 109563.3 | 267159.8 | 0.003 | 0.001 |
| 1\|per subject/paradigm | paradigm | 109569.7 | 267197.7 | 0.000 | 0.000 |
| 1\|per subject/paradigm | paradigm+offer | 109551.9 | 267147.9 | 1.000 | 0.522 |
| 1\|per subject/paradigm | paradigm*offer | 109554.7 | 267146.6 | 0.247 | 1.000 |

*Note: Green marks the best and simplest model.*

Table S4: Effects on offer for the P3.

|  | ***linked mastoid reference*** | | | | ***CSD reference*** | | | |
| --- | --- | --- | --- | --- | --- | --- | --- | --- |
| **offer differences** | **Estimate** | **Std. Error** | **z-value** | ***p*-value** | **Estimate** | **Std. Error** | **z-value** | ***p*-value** |
| 2-1 | -0.47 | 0.16 | -3.06 | 0.027 | -0.06 | 0.02 | -3.38 | 0.010 |
| 3-1 | -0.69 | 0.16 | -4.44 | <0.001 | -0.05 | 0.02 | -2.85 | 0.050 |
| 4-1 | -1.12 | 0.15 | -7.26 | <0.001 | -0.09 | 0.02 | -4.91 | <0.001 |
| 5-1 | -0.82 | 0.15 | -5.30 | <0.001 | -0.04 | 0.02 | -2.10 | 0.289 |
| 6-1 | -0.49 | 0.15 | -3.17 | 0.019 | -0.03 | 0.02 | -1.63 | 0.582 |
| 3-2 | -0.21 | 0.16 | -1.38 | 0.740 | 0.01 | 0.02 | 0.53 | 0.995 |
| 4-2 | -0.65 | 0.15 | -4.19 | <0.001 | -0.03 | 0.02 | -1.53 | 0.644 |
| 5-2 | -0.35 | 0.15 | -2.23 | 0.222 | 0.02 | 0.02 | 1.28 | 0.794 |
| 6-2 | -0.02 | 0.15 | -0.11 | 1 | 0.03 | 0.02 | 1.75 | 0.500 |
| 4-3 | -0.44 | 0.15 | -2.81 | 0.056 | -0.04 | 0.02 | -2.06 | 0.310 |
| 5-3 | -0.13 | 0.15 | -0.85 | 0.957 | 0.01 | 0.02 | 0.76 | 0.974 |
| 6-3 | 0.20 | 0.16 | 1.27 | 0.801 | 0.02 | 0.02 | 1.23 | 0.824 |
| 5-4 | 0.30 | 0.15 | 1.96 | 0.365 | 0.05 | 0.02 | 2.82 | 0.055 |
| 6-4 | 0.63 | 0.15 | 4.09 | <0.001 | 0.06 | 0.02 | 3.29 | 0.013 |
| 6-5 | 0.33 | 0.15 | 2.13 | 0.273 | 0.01 | 0.02 | 0.47 | 0.997 |

Table S5: Fit indices (AICc) and p-information loss for the midfrontal theta band response.

| **random effect** | **fixed effect** | **AIC  CSD reference** | **AIC  mastoid reference** | **Probability of no information loss to best model overall CSD reference** | **Probability of no information loss to best model overall mastoid reference** |
| --- | --- | --- | --- | --- | --- |
| 1\|per subject/paradigm |  | 236095.7 | 234233.5 | 0.000 | 0.000 |
| 1\|per subject/paradigm | offer | 236076.5 | 234223.2 | 0.000 | 0.000 |
| 1\|per subject/paradigm | paradigm | 236071.1 | 234206.5 | 0.000 | 0.004 |
| 1\|per subject/paradigm | paradigm+offer | 236051.9 | 234196.2 | 0.047 | 0.705 |
| 1\|per subject/paradigm | paradigm*offer | 236045.8 | 234195.5 | 1.000 | 1.000 |

*Note: Green marks the best and simplest model.*

Table S6: Effects on offer for the midfrontal theta band response.

|  | ***linked mastoid reference*** | | | | ***CSD reference*** | | | |
| --- | --- | --- | --- | --- | --- | --- | --- | --- |
| **offer differences** | **Estimate** | **Std. Error** | **z-value** | ***p*-value** | **Estimate** | **Std. Error** | **z-value** | ***p*-value** |
| 2-1 | 0.02 | 0.10 | 0.25 | 1.000 | -0.02 | 0.10 | -0.22 | 1.000 |
| 3-1 | 0.20 | 0.10 | 2.00 | 0.344 | 0.02 | 0.10 | 0.19 | 1.000 |
| 4-1 | 0.07 | 0.10 | 0.72 | 0.980 | 0.01 | 0.10 | 0.09 | 1.000 |
| 5-1 | -0.04 | 0.10 | -0.35 | 0.999 | -0.25 | 0.10 | -2.49 | 0.128 |
| 6-1 | -0.23 | 0.10 | -2.34 | 0.179 | -0.40 | 0.10 | -3.86 | 0.002 |
| 3-2 | 0.18 | 0.10 | 1.75 | 0.498 | 0.04 | 0.10 | 0.41 | 0.999 |
| 4-2 | 0.05 | 0.10 | 0.47 | 0.997 | 0.03 | 0.10 | 0.31 | 1.000 |
| 5-2 | -0.06 | 0.10 | -0.60 | 0.991 | -0.23 | 0.10 | -2.27 | 0.209 |
| 6-2 | -0.26 | 0.10 | -2.58 | 0.102 | -0.37 | 0.10 | -3.64 | 0.004 |
| 4-3 | -0.13 | 0.10 | -1.28 | 0.795 | -0.01 | 0.10 | -0.10 | 1.000 |
| 5-3 | -0.23 | 0.10 | -2.35 | 0.174 | -0.27 | 0.10 | -2.68 | 0.080 |
| 6-3 | -0.43 | 0.10 | -4.33 | <0.001 | -0.41 | 0.10 | -4.05 | <0.001 |
| 5-4 | -0.11 | 0.10 | -1.07 | 0.893 | -0.26 | 0.10 | -2.58 | 0.103 |
| 6-4 | -0.31 | 0.10 | -3.06 | 0.027 | -0.40 | 0.10 | -3.96 | 0.001 |
| 6-5 | -0.20 | 0.10 | -1.99 | 0.350 | -0.14 | 0.10 | -1.38 | 0.739 |

Table S7: Effects for best fitting model concerning midfrontal theta responses.

|  | ***linked mastoid reference*** | | | | | ***CSD reference*** | | | | |
| --- | --- | --- | --- | --- | --- | --- | --- | --- | --- | --- |
|  | **Estimate** | **Std. Error** | **z-value** | ***p*-value** | **robustness of significance** | **Estimate** | **Std. Error** | **z-value** | ***p*-value** | **robustness of significance** |
| (Intercept) | -0.93 | 0.13 | -7.04 | 0.000 | robust | -0.68 | 0.16 | -4.29 | 0.000 | robust |
| paradigm UG | 0.52 | 0.09 | 5.78 | 0.000 | robust | 0.83 | 0.17 | 4.84 | 0.000 | robust |
| offer2 | 0.02 | 0.10 | 0.25 | 0.806 | not significant | 0.06 | 0.14 | 0.39 | 0.697 | not significant |
| offer3 | 0.20 | 0.10 | 2.00 | 0.046 | exploratory | 0.18 | 0.14 | 1.24 | 0.216 | not significant |
| offer4 | 0.07 | 0.10 | 0.72 | 0.473 | not significant | -0.04 | 0.14 | -0.31 | 0.759 | not significant |
| offer5 | -0.04 | 0.10 | -0.35 | 0.725 | not significant | 0.01 | 0.14 | 0.06 | 0.949 | not significant |
| offer6 | -0.23 | 0.10 | -2.34 | 0.020 | exploratory | -0.15 | 0.14 | -1.04 | 0.300 | not significant |
| paradigm UG x offer2 |  |  |  |  |  | -0.16 | 0.20 | -0.77 | 0.442 | not significant |
| paradigm UG x offer3 |  |  |  |  |  | -0.32 | 0.20 | -1.56 | 0.120 | not significant |
| paradigm UG x offer4 |  |  |  |  |  | 0.11 | 0.20 | 0.52 | 0.603 | not significant |
| paradigm UG x offer5 |  |  |  |  |  | -0.53 | 0.20 | -2.57 | 0.010 | exploratory |
| paradigm UG x offer6 |  |  |  |  |  | -0.49 | 0.20 | -2.40 | 0.016 | exploratory |

Table S8: Fit indices (AICc) and p-information loss for the behavioral response.

| **random effect** | **fixed effect** | **AIC  CSD reference** | **AIC  mastoid reference** | **Probability of no information loss to best model overall CSD reference** | **Probability of no information loss to best model overall mastoid reference** | **conditional R² CSD reference** | **marginal R² CSD reference** | **conditional R² mastoid reference** | **marginal R² mastoid reference** |
| --- | --- | --- | --- | --- | --- | --- | --- | --- | --- |
| 1\|per subject |  | 16855.92 | 16855.92 | 0.000 | 0.000 | 0.64 | 0.00 | 0.64 | 0.00 |
| 1\|per subject | offer | 7937.412 | 7937.412 | 0.000 | 0.000 | 0.90 | 0.33 | 0.90 | 0.33 |
| 1\|per subject | offer + FRN per trial + mean FRN per subject | 7939.022 | 7939.977 | 0.000 | 0.000 | 0.90 | 0.34 | 0.90 | 0.34 |
| 1\|per subject | offer + P3 per trial + mean P3 per subject | 7922.381 | 7924.815 | 0.000 | 0.000 | 0.90 | 0.41 | 0.90 | 0.40 |
| 1\|per subject | offer + MFT per trial + mean MFT per subject | 7920.607 | 7927.902 | 0.000 | 0.000 | 0.90 | 0.43 | 0.90 | 0.39 |
| 1\|per subject | offer + FRN per trial + mean FRN per subject + P3 per trial + mean P3 per subject + MFT per trial + mean MFT per subject | 7914.246 | 7920.968 | 0.000 | 0.000 | 0.90 | 0.48 | 0.90 | 0.44 |
| 1\|per subject | offer * FRN per trial * mean FRN per subject | 7956.206 | 7931.032 | 0.000 | 0.000 | 0.90 | 0.34 | 0.90 | 0.34 |
| 1\|per subject | offer * P3 per trial * mean P3 per subject | 7913.464 | 7911.586 | 0.000 | 0.000 | 0.90 | 0.40 | 0.90 | 0.39 |
| 1\|per subject | offer * MFT per trial * mean MFT per subject | 7813.218 | 7862.887 | 1.000 | 1.000 | 0.90 | 0.43 | 0.90 | 0.38 |
| 1\|per subject | offer + FRN per trial * P3 per trial * MFT per trial * mean FRN per subject + FRN per trial * P3 per trial * MFT per trial * mean P3 per subject + FRN per trial * P3 per trial * MFT per trial * mean MFT per subject | not converged | 7936.2 |  | 0.000 |  |  | 0.91 | 0.47 |
|  |  |  |  |  |  |  |  |  |  |
| 1\|per subject | offer * MFT per trial * mean MFT per subject + offer * MFT per trial * acceptance button condition | 7767.1 | 7817.1 | 0.000 | 0.000 | 0.90 | 0.44 | 0.90 | 0.38 |
| 1\|per subject | offer * valence rating of trial type * MFT per trial * mean MFT per subject +offer * valence rating of trial type * MFT per trial * mean valence rating per subject +offer * valence rating of trial type * MFT per trial * acceptance button condition | 7529.7 | 7562.9 | 0.000 | 0.000 | 0.91 | 0.57 | 0.91 | 0.52 |
| 1\|per subject | offer * valence rating of trial type * MFT per trial * mean MFT per subject * mean valence rating per subject * acceptance button condition | 7429.1 | 7452.4 | 1.000 | 1.000 | 0.93 | 0.66 | 0.93 | 0.58 |

*Note: Light green marks the best and simplest model. Dark green marks the best model.*

Table S9: Effects for best fitting model concerning behavioral responses.

|  | ***linked mastoid reference*** | | | | | ***CSD reference*** | | | | |
| --- | --- | --- | --- | --- | --- | --- | --- | --- | --- | --- |
|  | **Estimate** | **Std. Error** | **z-value** | ***p*-value** | **robustness of significance** | **Estimate** | **Std. Error** | **z-value** | ***p*-value** | **robustness of significance** |
| (Intercept) | -1.49 | 0.43 | -3.47 | 0.001 | robust | -1.56 | 0.41 | -3.79 | 0.000 | robust |
| offer2 | 2.12 | 0.11 | 19.62 | 0.000 | robust | 2.19 | 0.11 | 19.63 | 0.000 | robust |
| offer3 | 4.00 | 0.12 | 32.49 | 0.000 | robust | 4.05 | 0.13 | 32.02 | 0.000 | robust |
| offer4 | 5.93 | 0.14 | 41.96 | 0.000 | robust | 5.89 | 0.14 | 41.11 | 0.000 | robust |
| offer5 | 7.99 | 0.17 | 46.04 | 0.000 | robust | 7.90 | 0.17 | 45.83 | 0.000 | robust |
| offer6 | 9.55 | 0.22 | 42.77 | 0.000 | robust | 9.68 | 0.23 | 41.71 | 0.000 | robust |
| MFT per trial | -0.01 | 0.01 | -0.46 | 0.646 | not significant | -0.01 | 0.01 | -0.45 | 0.651 | not significant |
| mean MFT per subject | -1.59 | 0.37 | -4.33 | 0.000 | robust | -2.22 | 0.33 | -6.65 | 0.000 | robust |
| offer2 x MFT per trial | 0.01 | 0.02 | 0.35 | 0.725 | not significant | 0.01 | 0.02 | 0.79 | 0.427 | not significant |
| offer3 x MFT per trial | 0.00 | 0.02 | 0.15 | 0.880 | not significant | 0.01 | 0.02 | 0.40 | 0.693 | not significant |
| offer4 x MFT per trial | -0.01 | 0.02 | -0.45 | 0.651 | not significant | 0.00 | 0.02 | 0.01 | 0.992 | not significant |
| offer5 x MFT per trial | -0.03 | 0.02 | -1.25 | 0.210 | not significant | -0.03 | 0.02 | -1.14 | 0.253 | not significant |
| offer6 x MFT per trial | -0.08 | 0.03 | -2.79 | 0.005 | exploratory | -0.10 | 0.03 | -3.37 | 0.001 | robust |
| offer2 x mean MFT per subject | 0.34 | 0.11 | 3.19 | 0.001 | robust | 0.68 | 0.10 | 6.53 | 0.000 | robust |
| offer3 x mean MFT per subject | 0.57 | 0.12 | 4.94 | 0.000 | robust | 0.90 | 0.11 | 8.19 | 0.000 | robust |
| offer4 x mean MFT per subject | 0.62 | 0.13 | 4.88 | 0.000 | robust | 1.17 | 0.12 | 9.69 | 0.000 | robust |
| offer5 x mean MFT per subject | 0.73 | 0.15 | 4.96 | 0.000 | robust | 1.33 | 0.14 | 9.65 | 0.000 | robust |
| offer6 x mean MFT per subject | 1.38 | 0.18 | 7.55 | 0.000 | robust | 1.50 | 0.17 | 9.07 | 0.000 | robust |
| MFT per trial x mean MFT per subject | 0.04 | 0.01 | 3.20 | 0.001 | robust | 0.01 | 0.01 | 0.48 | 0.632 | not significant |
| offer2 x MFT per trial x mean MFT per subject | -0.07 | 0.02 | -3.87 | 0.000 | robust | -0.01 | 0.02 | -0.63 | 0.530 | not significant |
| offer3 x MFT per trial x mean MFT per subject | -0.03 | 0.02 | -1.88 | 0.059 | not significant | 0.00 | 0.02 | 0.29 | 0.770 | not significant |
| offer4 x MFT per trial x mean MFT per subject | -0.03 | 0.02 | -1.91 | 0.057 | not significant | 0.00 | 0.02 | -0.13 | 0.895 | not significant |
| offer5 x MFT per trial x mean MFT per subject | -0.01 | 0.02 | -0.55 | 0.583 | not significant | 0.02 | 0.02 | 0.96 | 0.336 | not significant |
| offer6 x MFT per trial x mean MFT per subject | -0.06 | 0.02 | -2.36 | 0.018 | exploratory | 0.00 | 0.02 | 0.13 | 0.896 | not significant |

Table S10 Effects for best fitting model concerning behavioral responses with acceptance button positions.

|  | ***linked mastoid reference*** | | | | | ***CSD reference*** | | | | |
| --- | --- | --- | --- | --- | --- | --- | --- | --- | --- | --- |
|  | **Estimate** | **Std. Error** | **z-value** | ***p*-value** | **robustness of significance** | **Estimate** | **Std. Error** | **z-value** | ***p*-value** | **robustness of significance** |
| (Intercept) | -0.60 | 0.53 | -1.15 | 0.251 | not significant | -0.53 | 0.51 | -1.05 | 0.293 | not significant |
| offer2 | 1.45 | 0.37 | 3.92 | 0.000 | robust | 1.63 | 0.39 | 4.13 | 0.000 | robust |
| offer3 | 3.34 | 0.36 | 9.26 | 0.000 | robust | 3.14 | 0.39 | 8.13 | 0.000 | robust |
| offer4 | 5.38 | 0.38 | 14.28 | 0.000 | robust | 5.15 | 0.40 | 12.83 | 0.000 | robust |
| offer5 | 8.62 | 0.63 | 13.58 | 0.000 | robust | 8.18 | 0.57 | 14.28 | 0.000 | robust |
| offer6 | 7.58 | 1.41 | 5.36 | 0.000 | robust | 7.83 | 1.87 | 4.18 | 0.000 | robust |
| valence rating of trial type | 0.35 | 0.15 | 2.37 | 0.018 | exploratory | 0.48 | 0.16 | 3.00 | 0.003 | exploratory |
| mean valence rating per subject | 5.36 | 0.98 | 5.46 | 0.000 | robust | 6.48 | 1.02 | 6.38 | 0.000 | robust |
| MFT per trial | 0.02 | 0.04 | 0.47 | 0.635 | not significant | 0.01 | 0.05 | 0.13 | 0.893 | not significant |
| mean MFT per subject | -0.87 | 0.46 | -1.87 | 0.061 | not significant | -2.48 | 0.44 | -5.67 | 0.000 | robust |
| acceptance button position | -0.26 | 1.05 | -0.25 | 0.807 | not significant | -0.05 | 1.00 | -0.05 | 0.960 | not significant |
| offer2 * valence rating of trial type | -0.24 | 0.19 | -1.22 | 0.221 | not significant | -0.13 | 0.21 | -0.61 | 0.541 | not significant |
| offer3 * valence rating of trial type | -0.39 | 0.22 | -1.80 | 0.073 | not significant | -0.56 | 0.24 | -2.31 | 0.021 | exploratory |
| offer4 * valence rating of trial type | 0.63 | 0.24 | 2.63 | 0.009 | exploratory | 0.51 | 0.26 | 1.96 | 0.050 | exploratory |
| offer5 * valence rating of trial type | -0.38 | 0.45 | -0.84 | 0.400 | not significant | -0.42 | 0.39 | -1.07 | 0.284 | not significant |
| offer6 * valence rating of trial type | 0.41 | 0.49 | 0.84 | 0.400 | not significant | 0.08 | 0.63 | 0.12 | 0.905 | not significant |
| offer2 * mean valence rating per subject | -1.82 | 0.88 | -2.06 | 0.039 | exploratory | -2.93 | 0.93 | -3.14 | 0.002 | exploratory |
| offer3 * mean valence rating per subject | -2.58 | 0.88 | -2.95 | 0.003 | exploratory | -3.82 | 0.95 | -4.04 | 0.000 | robust |
| offer4 * mean valence rating per subject | -2.46 | 0.89 | -2.78 | 0.005 | exploratory | -3.73 | 0.95 | -3.90 | 0.000 | robust |
| offer5 * mean valence rating per subject | -0.47 | 1.09 | -0.43 | 0.669 | not significant | -1.76 | 1.16 | -1.52 | 0.129 | not significant |
| offer6 * mean valence rating per subject | -1.29 | 1.63 | -0.79 | 0.430 | not significant | -2.69 | 2.01 | -1.34 | 0.180 | not significant |
| valence rating of trial type * mean valence rating per subject | 1.03 | 0.33 | 3.16 | 0.002 | exploratory | 1.56 | 0.35 | 4.49 | 0.000 | robust |
| offer2 * MFT per trial | -0.01 | 0.05 | -0.27 | 0.787 | not significant | 0.00 | 0.05 | 0.01 | 0.994 | not significant |
| offer3 * MFT per trial | -0.02 | 0.04 | -0.51 | 0.612 | not significant | -0.01 | 0.05 | -0.10 | 0.918 | not significant |
| offer4 * MFT per trial | -0.06 | 0.04 | -1.37 | 0.169 | not significant | -0.02 | 0.05 | -0.35 | 0.725 | not significant |
| offer5 * MFT per trial | -0.04 | 0.09 | -0.43 | 0.665 | not significant | -0.02 | 0.08 | -0.26 | 0.793 | not significant |
| offer6 * MFT per trial | 0.06 | 0.20 | 0.29 | 0.774 | not significant | -0.37 | 0.22 | -1.70 | 0.089 | not significant |
| valence rating of trial type * MFT per trial | 0.00 | 0.02 | 0.29 | 0.769 | not significant | 0.00 | 0.02 | 0.18 | 0.859 | not significant |
| mean valence rating per subject * MFT per trial | -0.05 | 0.10 | -0.48 | 0.631 | not significant | -0.12 | 0.11 | -1.09 | 0.277 | not significant |
| offer2 * mean MFT per subject | -0.73 | 0.35 | -2.07 | 0.038 | exploratory | 0.29 | 0.40 | 0.74 | 0.458 | not significant |
| offer3 * mean MFT per subject | -0.53 | 0.34 | -1.58 | 0.114 | not significant | 0.58 | 0.39 | 1.49 | 0.137 | not significant |
| offer4 * mean MFT per subject | -0.32 | 0.35 | -0.94 | 0.349 | not significant | 1.09 | 0.40 | 2.73 | 0.006 | exploratory |
| offer5 * mean MFT per subject | -1.31 | 0.58 | -2.24 | 0.025 | exploratory | 0.33 | 0.54 | 0.61 | 0.540 | not significant |
| offer6 * mean MFT per subject | 0.60 | 0.94 | 0.63 | 0.527 | not significant | 1.80 | 1.22 | 1.48 | 0.140 | not significant |
| valence rating of trial type * mean MFT per subject | 0.18 | 0.14 | 1.29 | 0.199 | not significant | -0.17 | 0.17 | -1.00 | 0.320 | not significant |
| mean valence rating per subject * mean MFT per subject | 2.90 | 0.64 | 4.50 | 0.000 | robust | 3.09 | 0.65 | 4.78 | 0.000 | robust |
| MFT per trial * mean MFT per subject | 0.08 | 0.04 | 2.39 | 0.017 | exploratory | 0.06 | 0.05 | 1.33 | 0.185 | not significant |
| offer2 * acceptance button position | 1.99 | 0.74 | 2.69 | 0.007 | exploratory | 1.92 | 0.79 | 2.43 | 0.015 | exploratory |
| offer3 * acceptance button position | 1.23 | 0.71 | 1.72 | 0.086 | not significant | 1.14 | 0.77 | 1.47 | 0.140 | not significant |
| offer4 * acceptance button position | 1.12 | 0.74 | 1.50 | 0.133 | not significant | 1.10 | 0.80 | 1.38 | 0.169 | not significant |
| offer5 * acceptance button position | 2.35 | 1.26 | 1.86 | 0.062 | not significant | 1.56 | 1.14 | 1.37 | 0.171 | not significant |
| offer6 * acceptance button position | -1.42 | 2.83 | -0.50 | 0.616 | not significant | -1.49 | 3.74 | -0.40 | 0.690 | not significant |
| valence rating of trial type * acceptance button position | -0.26 | 0.29 | -0.91 | 0.364 | not significant | -0.31 | 0.32 | -0.97 | 0.332 | not significant |
| mean valence rating per subject * acceptance button position | -5.18 | 1.98 | -2.62 | 0.009 | exploratory | -3.91 | 2.04 | -1.92 | 0.055 | not significant |
| MFT per trial * acceptance button position | -0.15 | 0.08 | -1.88 | 0.060 | not significant | -0.17 | 0.09 | -1.90 | 0.058 | not significant |
| mean MFT per subject * acceptance button position | 2.52 | 0.93 | 2.72 | 0.007 | exploratory | -0.19 | 0.88 | -0.22 | 0.828 | not significant |
| offer2 * valence rating of trial type * mean valence rating per subject | -0.41 | 0.38 | -1.06 | 0.289 | not significant | -0.74 | 0.40 | -1.84 | 0.066 | not significant |
| offer3 * valence rating of trial type * mean valence rating per subject | -0.84 | 0.41 | -2.03 | 0.043 | exploratory | -1.47 | 0.44 | -3.33 | 0.001 | exploratory |
| offer4 * valence rating of trial type * mean valence rating per subject | -0.43 | 0.45 | -0.95 | 0.340 | not significant | -0.94 | 0.52 | -1.82 | 0.069 | not significant |
| offer5 * valence rating of trial type * mean valence rating per subject | -2.14 | 0.79 | -2.73 | 0.006 | exploratory | -2.66 | 0.80 | -3.33 | 0.001 | exploratory |
| offer6 * valence rating of trial type * mean valence rating per subject | -1.46 | 0.71 | -2.06 | 0.039 | exploratory | -2.11 | 0.80 | -2.64 | 0.008 | exploratory |
| offer2 * valence rating of trial type * MFT per trial | 0.00 | 0.03 | -0.12 | 0.907 | not significant | 0.01 | 0.03 | 0.26 | 0.798 | not significant |
| offer3 * valence rating of trial type * MFT per trial | 0.01 | 0.03 | 0.41 | 0.683 | not significant | 0.01 | 0.03 | 0.18 | 0.858 | not significant |
| offer4 * valence rating of trial type * MFT per trial | 0.04 | 0.03 | 1.32 | 0.189 | not significant | -0.02 | 0.03 | -0.47 | 0.641 | not significant |
| offer5 * valence rating of trial type * MFT per trial | -0.04 | 0.06 | -0.67 | 0.505 | not significant | -0.01 | 0.06 | -0.09 | 0.925 | not significant |
| offer6 * valence rating of trial type * MFT per trial | -0.07 | 0.06 | -1.12 | 0.265 | not significant | 0.08 | 0.07 | 1.03 | 0.303 | not significant |
| offer2 * mean valence rating per subject * MFT per trial | 0.12 | 0.11 | 1.12 | 0.264 | not significant | 0.16 | 0.12 | 1.38 | 0.169 | not significant |
| offer3 * mean valence rating per subject * MFT per trial | 0.00 | 0.10 | -0.01 | 0.992 | not significant | 0.10 | 0.11 | 0.88 | 0.377 | not significant |
| offer4 * mean valence rating per subject * MFT per trial | -0.02 | 0.10 | -0.23 | 0.817 | not significant | 0.09 | 0.11 | 0.77 | 0.441 | not significant |
| offer5 * mean valence rating per subject * MFT per trial | 0.04 | 0.13 | 0.29 | 0.774 | not significant | 0.21 | 0.14 | 1.46 | 0.145 | not significant |
| offer6 * mean valence rating per subject * MFT per trial | 0.11 | 0.24 | 0.46 | 0.647 | not significant | -0.03 | 0.24 | -0.14 | 0.886 | not significant |
| valence rating of trial type * mean valence rating per subject * MFT per trial | -0.02 | 0.03 | -0.51 | 0.608 | not significant | -0.04 | 0.04 | -1.08 | 0.280 | not significant |
| offer2 * valence rating of trial type * mean MFT per subject | -0.40 | 0.20 | -1.99 | 0.046 | exploratory | -0.21 | 0.21 | -0.99 | 0.320 | not significant |
| offer3 * valence rating of trial type * mean MFT per subject | -0.62 | 0.23 | -2.68 | 0.007 | exploratory | -0.25 | 0.23 | -1.07 | 0.283 | not significant |
| offer4 * valence rating of trial type * mean MFT per subject | -0.42 | 0.27 | -1.59 | 0.113 | not significant | 0.27 | 0.27 | 0.99 | 0.323 | not significant |
| offer5 * valence rating of trial type * mean MFT per subject | -0.02 | 0.35 | -0.06 | 0.955 | not significant | 0.19 | 0.32 | 0.59 | 0.556 | not significant |
| offer6 * valence rating of trial type * mean MFT per subject | -0.72 | 0.35 | -2.05 | 0.041 | exploratory | -0.09 | 0.41 | -0.22 | 0.829 | not significant |
| offer2 * mean valence rating per subject * mean MFT per subject | -1.81 | 0.50 | -3.63 | 0.000 | exploratory | -2.22 | 0.57 | -3.87 | 0.000 | robust |
| offer3 * mean valence rating per subject * mean MFT per subject | -3.26 | 0.53 | -6.19 | 0.000 | robust | -3.67 | 0.59 | -6.24 | 0.000 | robust |
| offer4 * mean valence rating per subject * mean MFT per subject | -3.03 | 0.52 | -5.79 | 0.000 | robust | -3.37 | 0.58 | -5.78 | 0.000 | robust |
| offer5 * mean valence rating per subject * mean MFT per subject | -5.22 | 0.65 | -7.98 | 0.000 | robust | -5.51 | 0.68 | -8.14 | 0.000 | robust |
| offer6 * mean valence rating per subject * mean MFT per subject | -3.92 | 1.38 | -2.85 | 0.004 | exploratory | -5.06 | 1.33 | -3.81 | 0.000 | robust |
| valence rating of trial type * mean valence rating per subject * mean MFT per subject | 0.89 | 0.24 | 3.72 | 0.000 | robust | 1.36 | 0.26 | 5.22 | 0.000 | robust |
| offer2 * MFT per trial * mean MFT per subject | -0.13 | 0.05 | -2.83 | 0.005 | exploratory | -0.08 | 0.05 | -1.49 | 0.135 | not significant |
| offer3 * MFT per trial * mean MFT per subject | -0.07 | 0.04 | -1.74 | 0.082 | not significant | -0.05 | 0.05 | -1.03 | 0.305 | not significant |
| offer4 * MFT per trial * mean MFT per subject | -0.05 | 0.04 | -1.21 | 0.228 | not significant | -0.06 | 0.05 | -1.22 | 0.221 | not significant |
| offer5 * MFT per trial * mean MFT per subject | -0.08 | 0.07 | -1.15 | 0.249 | not significant | -0.05 | 0.07 | -0.66 | 0.507 | not significant |
| offer6 * MFT per trial * mean MFT per subject | -0.19 | 0.14 | -1.34 | 0.181 | not significant | 0.00 | 0.14 | -0.01 | 0.992 | not significant |
| valence rating of trial type * MFT per trial * mean MFT per subject | 0.03 | 0.02 | 1.98 | 0.048 | exploratory | 0.03 | 0.02 | 1.60 | 0.110 | not significant |
| mean valence rating per subject * MFT per trial * mean MFT per subject | -0.05 | 0.06 | -0.84 | 0.402 | not significant | -0.13 | 0.06 | -2.03 | 0.042 | exploratory |
| offer2 * valence rating of trial type * acceptance button position | 0.78 | 0.38 | 2.02 | 0.043 | exploratory | 0.91 | 0.41 | 2.20 | 0.028 | exploratory |
| offer3 * valence rating of trial type * acceptance button position | 0.10 | 0.43 | 0.23 | 0.815 | not significant | -0.21 | 0.49 | -0.43 | 0.669 | not significant |
| offer4 * valence rating of trial type * acceptance button position | -0.75 | 0.48 | -1.57 | 0.116 | not significant | -0.78 | 0.52 | -1.51 | 0.132 | not significant |
| offer5 * valence rating of trial type * acceptance button position | -0.35 | 0.90 | -0.38 | 0.701 | not significant | 0.08 | 0.78 | 0.10 | 0.918 | not significant |
| offer6 * valence rating of trial type * acceptance button position | 0.50 | 0.98 | 0.51 | 0.608 | not significant | 0.60 | 1.26 | 0.48 | 0.633 | not significant |
| offer2 * mean valence rating per subject * acceptance button position | 4.39 | 1.77 | 2.48 | 0.013 | exploratory | 3.04 | 1.86 | 1.64 | 0.102 | not significant |
| offer3 * mean valence rating per subject * acceptance button position | 5.49 | 1.77 | 3.10 | 0.002 | exploratory | 4.26 | 1.90 | 2.25 | 0.025 | exploratory |
| offer4 * mean valence rating per subject * acceptance button position | 6.45 | 1.79 | 3.60 | 0.000 | exploratory | 4.76 | 1.91 | 2.49 | 0.013 | exploratory |
| offer5 * mean valence rating per subject * acceptance button position | 7.88 | 2.20 | 3.59 | 0.000 | exploratory | 5.39 | 2.32 | 2.32 | 0.020 | exploratory |
| offer6 * mean valence rating per subject * acceptance button position | 9.53 | 3.27 | 2.92 | 0.004 | exploratory | 9.56 | 4.02 | 2.38 | 0.017 | exploratory |
| valence rating of trial type * mean valence rating per subject * acceptance button position | -1.84 | 0.66 | -2.80 | 0.005 | exploratory | -1.40 | 0.69 | -2.02 | 0.044 | exploratory |
| offer2 * MFT per trial * acceptance button position | 0.29 | 0.10 | 3.01 | 0.003 | exploratory | 0.21 | 0.11 | 1.97 | 0.048 | exploratory |
| offer3 * MFT per trial * acceptance button position | 0.18 | 0.08 | 2.14 | 0.033 | exploratory | 0.24 | 0.10 | 2.42 | 0.016 | exploratory |
| offer4 * MFT per trial * acceptance button position | 0.16 | 0.09 | 1.80 | 0.072 | not significant | 0.18 | 0.10 | 1.79 | 0.074 | not significant |
| offer5 * MFT per trial * acceptance button position | 0.16 | 0.17 | 0.91 | 0.365 | not significant | 0.18 | 0.17 | 1.07 | 0.286 | not significant |
| offer6 * MFT per trial * acceptance button position | 0.61 | 0.40 | 1.52 | 0.128 | not significant | -0.10 | 0.43 | -0.23 | 0.819 | not significant |
| valence rating of trial type * MFT per trial * acceptance button position | -0.06 | 0.03 | -1.77 | 0.077 | not significant | -0.06 | 0.04 | -1.59 | 0.112 | not significant |
| mean valence rating per subject * MFT per trial * acceptance button position | 0.38 | 0.19 | 1.99 | 0.046 | exploratory | 0.40 | 0.22 | 1.85 | 0.064 | not significant |
| offer2 * mean MFT per subject * acceptance button position | -2.71 | 0.71 | -3.83 | 0.000 | robust | -0.64 | 0.79 | -0.81 | 0.420 | not significant |
| offer3 * mean MFT per subject * acceptance button position | -3.20 | 0.68 | -4.72 | 0.000 | robust | -0.43 | 0.77 | -0.56 | 0.578 | not significant |
| offer4 * mean MFT per subject * acceptance button position | -2.51 | 0.69 | -3.62 | 0.000 | exploratory | 0.14 | 0.80 | 0.17 | 0.864 | not significant |
| offer5 * mean MFT per subject * acceptance button position | -3.56 | 1.16 | -3.06 | 0.002 | exploratory | -0.31 | 1.08 | -0.29 | 0.773 | not significant |
| offer6 * mean MFT per subject * acceptance button position | -3.86 | 1.89 | -2.04 | 0.041 | exploratory | -1.90 | 2.44 | -0.78 | 0.437 | not significant |
| valence rating of trial type * mean MFT per subject * acceptance button position | 0.65 | 0.27 | 2.37 | 0.018 | exploratory | -0.53 | 0.34 | -1.59 | 0.113 | not significant |
| mean valence rating per subject * mean MFT per subject * acceptance button position | -0.71 | 1.28 | -0.55 | 0.580 | not significant | -1.73 | 1.29 | -1.34 | 0.181 | not significant |
| MFT per trial * mean MFT per subject * acceptance button position | -0.13 | 0.07 | -1.80 | 0.073 | not significant | -0.21 | 0.09 | -2.36 | 0.018 | exploratory |
| offer2 * valence rating of trial type * mean valence rating per subject * MFT per trial | 0.10 | 0.05 | 2.17 | 0.030 | exploratory | 0.11 | 0.05 | 2.20 | 0.028 | exploratory |
| offer3 * valence rating of trial type * mean valence rating per subject * MFT per trial | -0.03 | 0.05 | -0.60 | 0.548 | not significant | 0.02 | 0.05 | 0.42 | 0.674 | not significant |
| offer4 * valence rating of trial type * mean valence rating per subject * MFT per trial | 0.07 | 0.06 | 1.16 | 0.245 | not significant | 0.04 | 0.06 | 0.61 | 0.543 | not significant |
| offer5 * valence rating of trial type * mean valence rating per subject * MFT per trial | 0.05 | 0.09 | 0.61 | 0.544 | not significant | -0.04 | 0.09 | -0.42 | 0.677 | not significant |
| offer6 * valence rating of trial type * mean valence rating per subject * MFT per trial | -0.03 | 0.10 | -0.25 | 0.804 | not significant | 0.09 | 0.10 | 0.92 | 0.358 | not significant |
| offer2 * valence rating of trial type * mean valence rating per subject * mean MFT per subject | 0.34 | 0.32 | 1.05 | 0.294 | not significant | -0.18 | 0.35 | -0.52 | 0.605 | not significant |
| offer3 * valence rating of trial type * mean valence rating per subject * mean MFT per subject | -1.47 | 0.39 | -3.73 | 0.000 | robust | -1.71 | 0.40 | -4.26 | 0.000 | robust |
| offer4 * valence rating of trial type * mean valence rating per subject * mean MFT per subject | -1.35 | 0.46 | -2.92 | 0.003 | exploratory | -1.43 | 0.39 | -3.65 | 0.000 | exploratory |
| offer5 * valence rating of trial type * mean valence rating per subject * mean MFT per subject | 0.24 | 0.50 | 0.47 | 0.636 | not significant | -0.16 | 0.43 | -0.37 | 0.713 | not significant |
| offer6 * valence rating of trial type * mean valence rating per subject * mean MFT per subject | -1.07 | 0.70 | -1.54 | 0.124 | not significant | -1.03 | 0.55 | -1.87 | 0.061 | not significant |
| offer2 * valence rating of trial type * MFT per trial * mean MFT per subject | -0.02 | 0.03 | -0.85 | 0.394 | not significant | -0.06 | 0.03 | -2.01 | 0.045 | exploratory |
| offer3 * valence rating of trial type * MFT per trial * mean MFT per subject | -0.07 | 0.03 | -2.22 | 0.027 | exploratory | -0.06 | 0.03 | -1.88 | 0.060 | not significant |
| offer4 * valence rating of trial type * MFT per trial * mean MFT per subject | -0.07 | 0.03 | -2.05 | 0.040 | exploratory | -0.02 | 0.03 | -0.67 | 0.500 | not significant |
| offer5 * valence rating of trial type * MFT per trial * mean MFT per subject | -0.01 | 0.04 | -0.20 | 0.840 | not significant | -0.03 | 0.05 | -0.68 | 0.495 | not significant |
| offer6 * valence rating of trial type * MFT per trial * mean MFT per subject | 0.03 | 0.04 | 0.61 | 0.544 | not significant | -0.07 | 0.05 | -1.43 | 0.153 | not significant |
| offer2 * mean valence rating per subject * MFT per trial * mean MFT per subject | 0.11 | 0.07 | 1.63 | 0.104 | not significant | 0.15 | 0.08 | 1.94 | 0.052 | not significant |
| offer3 * mean valence rating per subject * MFT per trial * mean MFT per subject | 0.07 | 0.06 | 1.07 | 0.287 | not significant | 0.11 | 0.07 | 1.62 | 0.105 | not significant |
| offer4 * mean valence rating per subject * MFT per trial * mean MFT per subject | 0.06 | 0.06 | 0.91 | 0.365 | not significant | 0.12 | 0.07 | 1.80 | 0.072 | not significant |
| offer5 * mean valence rating per subject * MFT per trial * mean MFT per subject | 0.05 | 0.08 | 0.62 | 0.535 | not significant | 0.12 | 0.08 | 1.40 | 0.163 | not significant |
| offer6 * mean valence rating per subject * MFT per trial * mean MFT per subject | 0.08 | 0.24 | 0.32 | 0.748 | not significant | 0.22 | 0.15 | 1.45 | 0.146 | not significant |
| valence rating of trial type * mean valence rating per subject * MFT per trial * mean MFT per subject | -0.03 | 0.03 | -0.98 | 0.327 | not significant | -0.05 | 0.03 | -1.83 | 0.068 | not significant |
| offer2 * valence rating of trial type * mean valence rating per subject * acceptance button position | 1.41 | 0.77 | 1.85 | 0.065 | not significant | 0.85 | 0.80 | 1.07 | 0.285 | not significant |
| offer3 * valence rating of trial type * mean valence rating per subject * acceptance button position | 2.16 | 0.83 | 2.61 | 0.009 | exploratory | 1.77 | 0.89 | 1.99 | 0.046 | exploratory |
| offer4 * valence rating of trial type * mean valence rating per subject * acceptance button position | 0.22 | 0.91 | 0.25 | 0.806 | not significant | 0.08 | 1.04 | 0.08 | 0.936 | not significant |
| offer5 * valence rating of trial type * mean valence rating per subject * acceptance button position | 0.13 | 1.57 | 0.09 | 0.932 | not significant | -0.06 | 1.60 | -0.04 | 0.972 | not significant |
| offer6 * valence rating of trial type * mean valence rating per subject * acceptance button position | 0.80 | 1.41 | 0.57 | 0.570 | not significant | -0.11 | 1.60 | -0.07 | 0.945 | not significant |
| offer2 * valence rating of trial type * MFT per trial * acceptance button position | 0.12 | 0.05 | 2.45 | 0.014 | exploratory | 0.05 | 0.06 | 0.90 | 0.367 | not significant |
| offer3 * valence rating of trial type * MFT per trial * acceptance button position | 0.18 | 0.06 | 3.07 | 0.002 | exploratory | 0.14 | 0.07 | 2.17 | 0.030 | exploratory |
| offer4 * valence rating of trial type * MFT per trial * acceptance button position | 0.04 | 0.06 | 0.69 | 0.491 | not significant | 0.07 | 0.07 | 1.05 | 0.292 | not significant |
| offer5 * valence rating of trial type * MFT per trial * acceptance button position | -0.08 | 0.12 | -0.65 | 0.517 | not significant | 0.07 | 0.13 | 0.60 | 0.552 | not significant |
| offer6 * valence rating of trial type * MFT per trial * acceptance button position | -0.07 | 0.13 | -0.53 | 0.597 | not significant | 0.22 | 0.15 | 1.48 | 0.138 | not significant |
| offer2 * mean valence rating per subject * MFT per trial * acceptance button position | -0.67 | 0.21 | -3.16 | 0.002 | exploratory | -0.51 | 0.23 | -2.21 | 0.027 | exploratory |
| offer3 * mean valence rating per subject * MFT per trial * acceptance button position | -0.40 | 0.20 | -2.00 | 0.046 | exploratory | -0.38 | 0.22 | -1.74 | 0.082 | not significant |
| offer4 * mean valence rating per subject * MFT per trial * acceptance button position | -0.41 | 0.20 | -2.06 | 0.039 | exploratory | -0.45 | 0.22 | -2.03 | 0.042 | exploratory |
| offer5 * mean valence rating per subject * MFT per trial * acceptance button position | -0.40 | 0.26 | -1.52 | 0.128 | not significant | -0.17 | 0.29 | -0.59 | 0.554 | not significant |
| offer6 * mean valence rating per subject * MFT per trial * acceptance button position | -0.13 | 0.47 | -0.26 | 0.792 | not significant | -0.76 | 0.47 | -1.61 | 0.108 | not significant |
| valence rating of trial type * mean valence rating per subject * MFT per trial * acceptance button position | 0.12 | 0.07 | 1.81 | 0.071 | not significant | 0.14 | 0.08 | 1.90 | 0.058 | not significant |
| offer2 * valence rating of trial type * mean MFT per subject * acceptance button position | -0.68 | 0.40 | -1.68 | 0.094 | not significant | -0.05 | 0.42 | -0.12 | 0.906 | not significant |
| offer3 * valence rating of trial type * mean MFT per subject * acceptance button position | -0.27 | 0.46 | -0.58 | 0.565 | not significant | 0.59 | 0.47 | 1.27 | 0.205 | not significant |
| offer4 * valence rating of trial type * mean MFT per subject * acceptance button position | -1.18 | 0.53 | -2.21 | 0.027 | exploratory | -0.40 | 0.55 | -0.74 | 0.461 | not significant |
| offer5 * valence rating of trial type * mean MFT per subject * acceptance button position | -0.51 | 0.70 | -0.73 | 0.467 | not significant | 0.58 | 0.64 | 0.90 | 0.369 | not significant |
| offer6 * valence rating of trial type * mean MFT per subject * acceptance button position | -0.26 | 0.70 | -0.37 | 0.712 | not significant | 0.73 | 0.81 | 0.91 | 0.364 | not significant |
| offer2 * mean valence rating per subject * mean MFT per subject * acceptance button position | -1.42 | 1.00 | -1.42 | 0.155 | not significant | 0.09 | 1.15 | 0.08 | 0.937 | not significant |
| offer3 * mean valence rating per subject * mean MFT per subject * acceptance button position | 1.16 | 1.05 | 1.11 | 0.269 | not significant | 3.22 | 1.17 | 2.75 | 0.006 | exploratory |
| offer4 * mean valence rating per subject * mean MFT per subject * acceptance button position | 0.63 | 1.04 | 0.61 | 0.545 | not significant | 2.61 | 1.16 | 2.24 | 0.025 | exploratory |
| offer5 * mean valence rating per subject * mean MFT per subject * acceptance button position | -0.41 | 1.30 | -0.31 | 0.755 | not significant | 2.63 | 1.35 | 1.94 | 0.052 | not significant |
| offer6 * mean valence rating per subject * mean MFT per subject * acceptance button position | 2.98 | 2.75 | 1.08 | 0.279 | not significant | 4.13 | 2.65 | 1.56 | 0.120 | not significant |
| valence rating of trial type * mean valence rating per subject * mean MFT per subject * acceptance button position | -0.18 | 0.48 | -0.37 | 0.713 | not significant | -1.38 | 0.52 | -2.64 | 0.008 | exploratory |
| offer2 * MFT per trial * mean MFT per subject * acceptance button position | 0.12 | 0.09 | 1.29 | 0.196 | not significant | 0.27 | 0.11 | 2.51 | 0.012 | exploratory |
| offer3 * MFT per trial * mean MFT per subject * acceptance button position | 0.09 | 0.08 | 1.15 | 0.252 | not significant | 0.17 | 0.10 | 1.76 | 0.079 | not significant |
| offer4 * MFT per trial * mean MFT per subject * acceptance button position | 0.16 | 0.09 | 1.86 | 0.062 | not significant | 0.20 | 0.10 | 2.03 | 0.042 | exploratory |
| offer5 * MFT per trial * mean MFT per subject * acceptance button position | 0.04 | 0.14 | 0.27 | 0.787 | not significant | 0.11 | 0.15 | 0.71 | 0.481 | not significant |
| offer6 * MFT per trial * mean MFT per subject * acceptance button position | 0.00 | 0.28 | -0.02 | 0.988 | not significant | 0.42 | 0.28 | 1.49 | 0.136 | not significant |
| valence rating of trial type * MFT per trial * mean MFT per subject * acceptance button position | -0.06 | 0.03 | -2.09 | 0.037 | exploratory | -0.11 | 0.04 | -2.61 | 0.009 | exploratory |
| mean valence rating per subject * MFT per trial * mean MFT per subject * acceptance button position | 0.25 | 0.12 | 2.12 | 0.034 | exploratory | 0.36 | 0.13 | 2.85 | 0.004 | exploratory |
| offer2 * valence rating of trial type * mean valence rating per subject * MFT per trial * mean MFT per subject | 0.11 | 0.04 | 2.54 | 0.011 | exploratory | 0.06 | 0.04 | 1.42 | 0.157 | not significant |
| offer3 * valence rating of trial type * mean valence rating per subject * MFT per trial * mean MFT per subject | -0.01 | 0.04 | -0.22 | 0.827 | not significant | 0.02 | 0.04 | 0.53 | 0.597 | not significant |
| offer4 * valence rating of trial type * mean valence rating per subject * MFT per trial * mean MFT per subject | -0.08 | 0.06 | -1.39 | 0.164 | not significant | 0.02 | 0.04 | 0.51 | 0.613 | not significant |
| offer5 * valence rating of trial type * mean valence rating per subject * MFT per trial * mean MFT per subject | 0.02 | 0.06 | 0.28 | 0.776 | not significant | 0.07 | 0.05 | 1.49 | 0.137 | not significant |
| offer6 * valence rating of trial type * mean valence rating per subject * MFT per trial * mean MFT per subject | 0.04 | 0.12 | 0.34 | 0.733 | not significant | 0.03 | 0.06 | 0.50 | 0.617 | not significant |
| offer2 * valence rating of trial type * mean valence rating per subject * MFT per trial * acceptance button position | -0.38 | 0.09 | -4.05 | 0.000 | robust | -0.30 | 0.10 | -2.98 | 0.003 | exploratory |
| offer3 * valence rating of trial type * mean valence rating per subject * MFT per trial * acceptance button position | -0.09 | 0.09 | -1.02 | 0.309 | not significant | -0.20 | 0.10 | -1.96 | 0.050 | not significant |
| offer4 * valence rating of trial type * mean valence rating per subject * MFT per trial * acceptance button position | -0.21 | 0.12 | -1.82 | 0.069 | not significant | -0.19 | 0.13 | -1.45 | 0.148 | not significant |
| offer5 * valence rating of trial type * mean valence rating per subject * MFT per trial * acceptance button position | -0.14 | 0.17 | -0.78 | 0.434 | not significant | -0.31 | 0.18 | -1.72 | 0.086 | not significant |
| offer6 * valence rating of trial type * mean valence rating per subject * MFT per trial * acceptance button position | -0.19 | 0.21 | -0.88 | 0.377 | not significant | 0.01 | 0.20 | 0.04 | 0.969 | not significant |
| offer2 * valence rating of trial type * mean valence rating per subject * mean MFT per subject * acceptance button position | -1.78 | 0.64 | -2.77 | 0.006 | exploratory | -0.39 | 0.70 | -0.56 | 0.579 | not significant |
| offer3 * valence rating of trial type * mean valence rating per subject * mean MFT per subject * acceptance button position | 2.14 | 0.79 | 2.72 | 0.007 | exploratory | 3.42 | 0.80 | 4.28 | 0.000 | robust |
| offer4 * valence rating of trial type * mean valence rating per subject * mean MFT per subject * acceptance button position | 1.37 | 0.93 | 1.48 | 0.138 | not significant | 1.65 | 0.78 | 2.11 | 0.035 | exploratory |
| offer5 * valence rating of trial type * mean valence rating per subject * mean MFT per subject * acceptance button position | 0.92 | 1.01 | 0.91 | 0.364 | not significant | 1.21 | 0.86 | 1.41 | 0.158 | not significant |
| offer6 * valence rating of trial type * mean valence rating per subject * mean MFT per subject * acceptance button position | -0.80 | 1.39 | -0.58 | 0.563 | not significant | 0.72 | 1.10 | 0.65 | 0.514 | not significant |
| offer2 * valence rating of trial type * MFT per trial * mean MFT per subject * acceptance button position | 0.00 | 0.06 | 0.05 | 0.964 | not significant | 0.16 | 0.06 | 2.79 | 0.005 | exploratory |
| offer3 * valence rating of trial type * MFT per trial * mean MFT per subject * acceptance button position | 0.00 | 0.06 | 0.05 | 0.959 | not significant | 0.06 | 0.06 | 0.99 | 0.322 | not significant |
| offer4 * valence rating of trial type * MFT per trial * mean MFT per subject * acceptance button position | 0.05 | 0.07 | 0.76 | 0.450 | not significant | 0.15 | 0.06 | 2.34 | 0.020 | exploratory |
| offer5 * valence rating of trial type * MFT per trial * mean MFT per subject * acceptance button position | 0.17 | 0.08 | 1.98 | 0.048 | exploratory | 0.13 | 0.09 | 1.43 | 0.152 | not significant |
| offer6 * valence rating of trial type * MFT per trial * mean MFT per subject * acceptance button position | 0.04 | 0.09 | 0.46 | 0.643 | not significant | 0.03 | 0.09 | 0.38 | 0.707 | not significant |
| offer2 * mean valence rating per subject * MFT per trial * mean MFT per subject * acceptance button position | -0.38 | 0.14 | -2.79 | 0.005 | exploratory | -0.40 | 0.15 | -2.63 | 0.008 | exploratory |
| offer3 * mean valence rating per subject * MFT per trial * mean MFT per subject * acceptance button position | -0.26 | 0.12 | -2.13 | 0.033 | exploratory | -0.39 | 0.13 | -2.89 | 0.004 | exploratory |
| offer4 * mean valence rating per subject * MFT per trial * mean MFT per subject * acceptance button position | -0.26 | 0.13 | -2.02 | 0.043 | exploratory | -0.38 | 0.13 | -2.84 | 0.004 | exploratory |
| offer5 * mean valence rating per subject * MFT per trial * mean MFT per subject * acceptance button position | -0.26 | 0.16 | -1.67 | 0.096 | not significant | -0.47 | 0.17 | -2.76 | 0.006 | exploratory |
| offer6 * mean valence rating per subject * MFT per trial * mean MFT per subject * acceptance button position | -0.39 | 0.47 | -0.84 | 0.403 | not significant | -0.43 | 0.30 | -1.43 | 0.153 | not significant |
| valence rating of trial type * mean valence rating per subject * MFT per trial * mean MFT per subject * acceptance button position | 0.12 | 0.05 | 2.32 | 0.021 | exploratory | 0.14 | 0.05 | 2.63 | 0.008 | exploratory |
| offer2 * valence rating of trial type * mean valence rating per subject * MFT per trial * mean MFT per subject * acceptance button position | -0.21 | 0.08 | -2.59 | 0.010 | exploratory | -0.12 | 0.09 | -1.37 | 0.170 | not significant |
| offer3 * valence rating of trial type * mean valence rating per subject * MFT per trial * mean MFT per subject * acceptance button position | -0.16 | 0.09 | -1.81 | 0.071 | not significant | -0.14 | 0.09 | -1.62 | 0.105 | not significant |
| offer4 * valence rating of trial type * mean valence rating per subject * MFT per trial * mean MFT per subject * acceptance button position | -0.17 | 0.12 | -1.42 | 0.156 | not significant | -0.17 | 0.09 | -1.98 | 0.048 | exploratory |
| offer5 * valence rating of trial type * mean valence rating per subject * MFT per trial * mean MFT per subject * acceptance button position | -0.11 | 0.11 | -1.00 | 0.316 | not significant | -0.09 | 0.10 | -0.91 | 0.362 | not significant |
| offer6 * valence rating of trial type * mean valence rating per subject * MFT per trial * mean MFT per subject * acceptance button position | -0.15 | 0.24 | -0.61 | 0.543 | not significant | -0.12 | 0.12 | -0.98 | 0.328 | not significant |

**Supplemental Material S11: Detailed topographical results for FRN and P3.**

# Results

### FRN

#### Anteriority

In all three analyses on anteriority (replication, extension_time-window,_ extension_CSD_), all positions were different from each other (replication: *F*(4,416) = 111.79, *p* < .001, $\eta_{p}^{2}$ = .52, GG = .28; extension_time-window_: *F*(4,416) = 95.10, *p* < .001, $\eta_{p}^{2}$ = .48, GG = .28; extension_CSD_: *F*(4,416) = 51.13, *p* < .001, $\eta_{p}^{2}$ = .33, GG = .67), revealing a gradient with the most negative values in frontal regions to the least negative values in the posterior regions (*p*s < .001).

#### Laterality

For replication and extension_time-window_, the lateral factor (replication: *F*(2,208) = 11.49, *p* < .001, $\eta_{p}^{2}$ = .10, GG = .95; extension_time-window_: *F*(2,208) = 14.19, *p* < .001, $\eta_{p}^{2}$ = .12, GG = .96) revealed that the values on the midline were most negative, that those on the right side were most positive, and that the left side fell in between (*p*s < .001). For extension_CSD_ (*F*(2,208) = 9.71, *p* < .001, $\eta_{p}^{2}$ = .09, GG = .80). The most negative FRN values were observed on the left side, followed by the right side. The midline showed the most positive FRN values (*p*s < .01).

#### Offer x Anteriority

For replication and extension_time-window_, the interaction offer x anterior (replication: *F*(20,2080) = 10.37, *p* < .001, $\eta_{p}^{2} =$.09, GG = .27; extension_time-window_: *F*(20,2080) = 12.09, *p* < .001, $\eta_{p}^{2}$ = .10, GG = .27) revealed that the patterns of the main effects for offers were mostly preserved in the frontal and frontocentral regions. In the replication analysis, there were no significant differences between the highest and lowest offer, the 2-credits and 3- or 4-credits offer, and the 5-credits and 6-credits offer (*p*s > .754). In the extension_time-window_, the FRN to 1-credit offers was still lower than to any other offer except for the highest offer in frontocentral regions (*p*s < .01) and the highest and second-highest offer in the frontal region (*p*s < .01). In the replication, for the central, central-posterior, as well as the posterior regions, the highest offer was not different from any other offer (*p*s = 1). While the difference between the lowest and the second-lowest offer declined in the central regions (*p* = .168), this decline was enlarged to the 3-credits offer in the central-posterior regions (*p*s > .082) until no significant difference could be detected among any offer in the posterior regions (*p*s > .062). In the extension_time-window_, the difference was present for all but the 2:10 and 6:6 offer in the central region (*p*s < .05). In central-posterior regions, the 3:9 and 5:7 offer were significantly more negative than the 1:11 offer (*p*s < .05). In addition, in the frontal region, the highest offer was significantly lower in FRN amplitude than any other offer, except for the lowest offer (*p*s < .05). In the frontal-central regions, the highest offer had a significantly less negative FRN than the two middle-offer categories 3:9 and 4:8 (*p*s < .05). In the extension_CSD_ analysis (*F*(20,2080) = 11.60, *p* < .001, $\eta_{p}^{2} =$.10, GG = .48), no significant differences for offers were found at any sites (*p*s > .079), except for central-posterior positions where the FRN amplitudes for 1, 2, and 3-credits offers were more positive than the those for 5 and 6-credits offers (*p*s < .05).

#### Offer x Laterality

The interaction offer x lateral was significant in all three analyses (replication: *F*(10,1040) = 12.10, *p* < .001, $\eta_{p}^{2}$ = .10, GG = .68; extension_time-window_: *F*(10,1040) = 12.06, *p* < .001, $\eta_{p}^{2}$ = .10, GG = .68; extension_CSD_: *F*(10,1040) = 4.67, *p* < .001, $\eta_{p}^{2} =$.04, GG = .79).

Post-hoc tests in the replication analysis indicated that, for the left and midline sites, the differences between the highest and the lowest offer declined, as did the difference between the second-highest offer and the highest offer along with the differences between the 2-credits offer and the 3 and 4-credits offers (*p* > .251). For the right sites, the only offer that differed from other offers except for the second lowest (*p* = .159) was the lowest offer (*p*s < .002).

In the extension_CSD_ analysis, for central sites, the lowest offer had a lower FRN than all other offers, except for the highest offer (*p*s < .001). For the left sites, a similar effect was shown (*p*s < .05), but the second-highest offer also did not differ from the lowest (*p* = .225). For the right sites, the lowest offer differed from all offers (*p*s < .05) except for the 2:10 offer (*p* = .40). In addition, the FRN for 3:9 offers was more negative than the FRN for the highest offer in left and central sites (*p*s < .001), and the FRN for 4:8 offers was more negative than the FRN for the highest offer (*p* < .05).

In the extension_CSD_ analysis, the only significant difference was found on the midline, with the 1-credit offer showing a more positive FRN than the 6-credits offer.

#### Anteriority x Laterality

The interaction anterior x lateral was significant in all three analyses (replication: *F*(8,832) = 6.31, *p* < .001, $\eta_{p}^{2}$ = .06, GG = .45; extension_time-window_: (*F*(8,832) = 7.10, *p* < .001, $\eta_{p}^{2}$= .06, GG = .44; extension_CSD_: *F*(8,832) = 57.47, *p* < .001, $\eta_{p}^{2}$= .36, GG = .36).

In the replication analysis, the interaction revealed the main-effect pattern of the anterior gradient for the central electrodes (*p*s < .01) as well as a decline of the difference in frontal and frontocentral regions on the left and right sites (*p*s = 1).

In the extension_time-window_ analysis, the main-effect pattern of the anterior gradient was found for all sites. However, the frontal and frontocentral cites were not significantly different (*p*s > .06) as they showed no detectable difference in the patterns (except for a marginally significant effect of the frontal and frontocentral sites on the right (*p* = .0515) compared to the other sites (*p*s = 1)). In the extension_CSD_ analysis, on both the left and right sites, the posterior signal was more positive than all other electrodes (*p*s < .001) and the central-posterior signal was more positive than the central-site signal (*p* < .05). For the right sites, the central-posterior signal was also more positive than the frontocentral signal (*p* < .001), while the frontal sites were more positive than the frontocentral and central sites (*p*s < .05). On the midline, all electrode positions differed from each other (*p*s < .001), except for the frontal electrode, which did not differ from the frontocentral electrode (*p* = 1). The revealed pattern showed the most positive values on the central-posterior electrodes, followed by the central electrodes, and then the posterior electrodes. The most negative signals were detected at frontal and frontocentral electrodes.

#### Offer x Anteriority x Laterality

In the replication analysis (*F*(40,4160) = 6.21, *p* < .001, $\eta_{p}^{2}$= .06, GG = .38), the threefold interaction offer x anterior x lateral revealed a decline of all offer differences except for the difference between the lowest offer and the 3-credits offer on frontocentral and frontocentral midline regions (*p*s < .034; see Figure S11a panel A).

In the extension_time-window_ analysis, the interaction (*F*(40,4160) = 4.92, *p* < .001, $\eta_{p}^{2}$= .05, GG = .37) could not be interpreted using post-hoc *t-*tests as all offer differences became non-significant (*p*s > .9), possibly due to the overly conservative correction of the Bonferroni-Holm method.

In the extension_CSD_ analysis, the threefold interaction (*F*(40,4160) = 15.16, *p* < .001, $\eta_{p}^{2}$= .13, GG = .31) showed that for the midline electrode at the central-posterior sites, the FRN signal for the 5 and 6-credits offers was more negative than for all other offers. On the right sites on the posterior positions, the signal was more positive for the 2-credits than the 6-credits offer (see Figure S11a panel B).


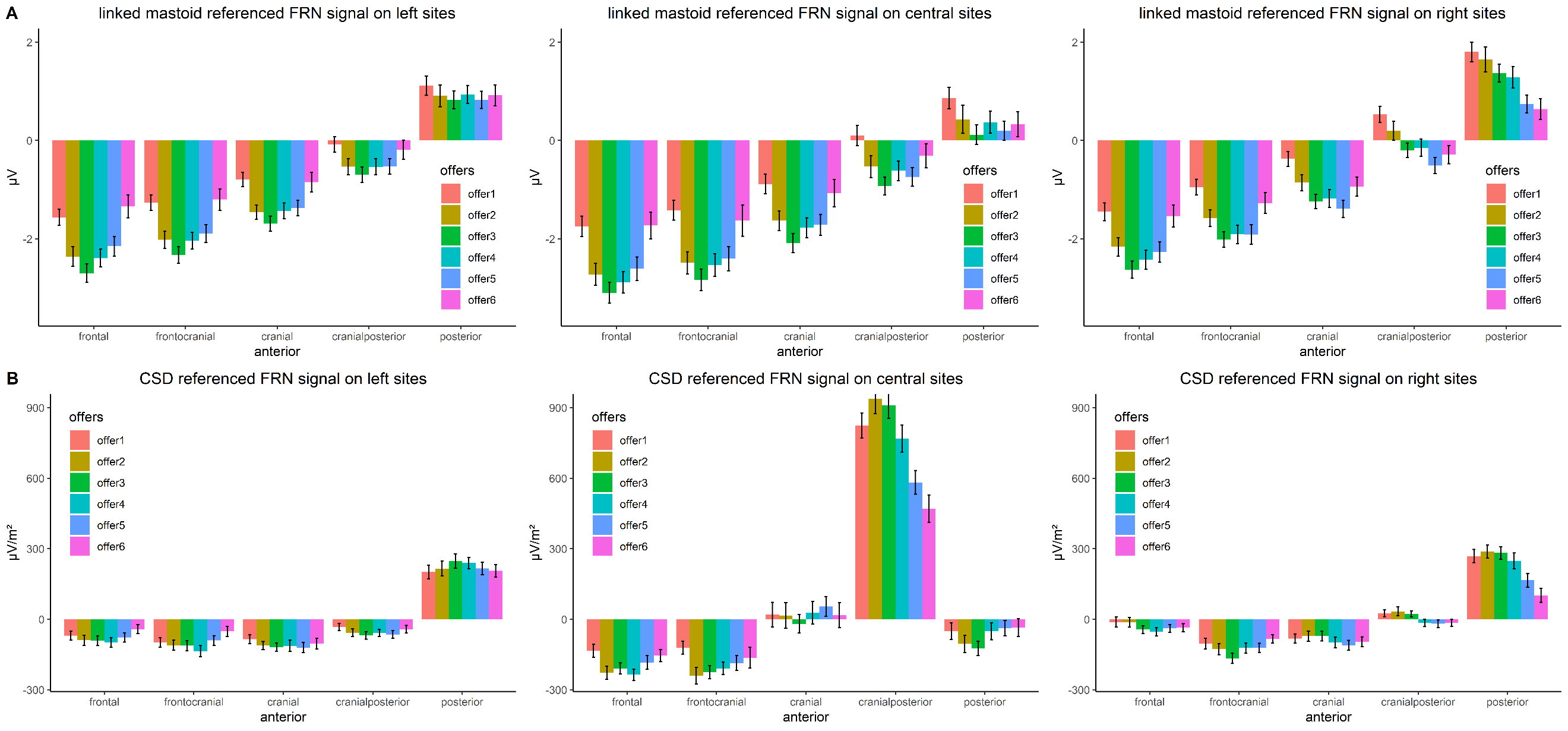


Figure S11a: A. Threefold interaction between, offer, anteriority, and laterality for FRN signal. B. The same threefold interaction with the new quantification CSD reference. Error-bars depict mean within SEM.

#### Paradigm x Anteriority x Laterality

In the replication analysis, the threefold interaction paradigm x anteriority x laterality (*F*(8,832) = 4.54, *p* < .001, $\eta_{p}^{2}$= .04, GG = .41) revealed that the frontal and frontocentral areas did not differ concerning the anterior gradient (*p*s > .071), except for the midline frontocentral area in the UG (*p* < .001). In contrast, in the extension_time-window_ analysis (*F*(8,832) = 3.24, *p* < .01, $\eta_{p}^{2}$= .03, GG = .43), the anterior gradient could be detected (*p*s < .05) except for a lack of difference between the frontal and the frontocentral sites (*p*s = 1). Moreover, for all but the midline sites in the UG (*p* = .023), the central sites did not differ from the frontocentral sites (*p*s > .380). In the extension_CSD_ analysis, the threefold interaction was not significant.

#### Offer x Paradigm x Anteriority

In the extension_time-window_ analysis, the threefold interaction offer x anterior x paradigm (*F*(20,2080) = 2.11, *p* < .01, $\eta_{p}^{2}$= .02) revealed that the DG did not show the effect patterns (*p*s = 1) except for the UG. Here, at frontal and frontocentral sites, the lowest offer had a more positive FRN than the 3:9, 4:8, and 5:7 offers (*p*s < .01). For central sites, only the 3:9 and 5:7 FRN were more negative than the 1:11 offer (*p*s < .05). For the highest offer, differences in FRN to the 5:7, 4:8, and 3:9 offers were observed at frontal sites, indicating a more positive FRN for the highest offer (*p*s < .05). For the frontocentral sites, this difference was only found for the 3:9 offer (*p* < .01).

Other interactions concerning FRN, including the fourfold interaction, were not significant.

### P3

#### Anteriority

In the analyses on anteriority, all positions were different (replication: *F*(4,416) = 45.22, *p* < .001, $\eta_{p}^{2}$= .30, GG = .30; extension_time-window_: *F*(4,416) = 97.94, *p* < .001, $\eta_{p}^{2}$= .49, GG = .29; extension_CSD_: *F*(4,416) = 47.25, *p* < .001, $\eta_{p}^{2}$= .31, GG = .68), except for the frontal values which did not significantly differ from the frontocentral activation in the extension_CSD_ analysis (*p* = .089). The resulting pattern revealed a gradient, with the most negative values observed in frontal regions and the least negative values in the posterior regions (*p*s < .001).

#### Laterality

The main effects for laterality in the replication (*F*(2,208) = 12.50, *p* < .001, $\eta_{p}^{2}$= .11, GG = .96) and the extension_time-window_ analyses (*F*(2,208) = 5.33, *p* < .001, $\eta_{p}^{2}$= .05, GG = .98) showed that the values on the left were significantly different from those on the right and the midline (*p*s < .001).

In the extension_CSD_ analysis (*F*(2,208) = 23.72, *p* < .001, $\eta_{p}^{2}$= .19, GG = .82), the values on the left were significantly lower than on the right (*p*s < .001), with the most positive values observed on the midline (*p*s < .001).

#### Offer x Anteriority

In the extension_time-window_ and the extension_CSD_ analyses but not the replication analysis, the interaction offer x anterior was significant (extension_time-window_: *F*(20,2080) = 4.10, *p* < .001, $\eta_{p}^{2}$= .04, GG = .25; extension_CSD_: *F*(20,2080) = 5.54, *p* < .001, $\eta_{p}^{2}$= .05, GG = .45). For extension_time-window_, the post-hoc test revealed that the lowest offer significantly differed from the 3, 4, and 5-credits offers in frontal and fronto-central positions (*p*s < .05). On central positions, the difference was only significant for 1-credit compared to 4-credits offers (*p* = .009). For extension_CSD_, post-hoc tests indicated that the P3 signal for the lowest offer was significantly higher than for to the 4-credits offer in frontal position (*p* < .05), and that the P3 signal for the 5 and 6-credits offers were lower than those for the 1, 2, and 3-credits offers (*p*s < .01).

#### Offer x Laterality

The interaction offer x lateral was significant for all three analyses, but the patterns differed from each other. In the replication analysis (*F*(10,1040) = 10.89, *p* < .001, $\eta_{p}^{2}$= .09, GG = .48), the post-hoc test revealed that the offer effects further declined on the left side, given that they were only present for the difference between the lowest and the two middle offers of 3 and 4 credits (*p*s < .05), and the difference between the highest and the 3-credits offer (*p* < .01). On the right side, the lowest offer differed from all other offers (*p*s < .01), while the 2-credits offer differed from the two middle offers (*p*s < .01). On the midline, the lowest offer only differed from the 3, 4, and 5-credits offers (*p*s < .001), the highest offer from the two middle categories (*p*s < .05), and the second-lowest offer from the lowest offer (*p* = .003).

In the extension_time-window_ analysis (*F*(10,1040) = 11.18, *p* < .001, $\eta_{p}^{2}$= .10, GG = .75), post-hoc tests revealed that the offer effects further declined on the left side, given that they were only present for the difference between the lowest offer and the 4-credits offer (*p* = .008). On the right side, the lowest offer was different from all other conditions (*p*s < .01) and the 2-credit offer differed from the 3 and 4-credits offers (*p*s < .05). On the midline, the lowest offer differed from all other offers (*p*s < .01) and the second-lowest offer differed from the 4-credits offer (*p* = .003).

In the extension_CSD_ analysis (*F*(10,1040) = 11.85, *p* < .001, $\eta_{p}^{2}$= .10, GG = .76), the post-hoc tests revealed a higher P3 signal for the lowest offer compared to the 4, 5, and 6-credits offers (*p*s < .001) as well as a lower P3 signal for the highest offer compared to the 1, 2, and 3-credits offers (*p*s < .05), but only on midline electrodes.

#### Anteriority x Laterality

The interaction between the anterior and the lateral factor was significant in all analyses. In the replication analysis (*F*(8,832) = 11.24, *p* < .001, $\eta_{p}^{2}$= .10, GG = .49), the anterior gradient could be detected except on the left side for the differences between the frontal and frontocentral section, the frontocentral to the central section, and the central to central-posterior section (*p*s > .06). On the midline, the gradient was present from the frontocentral to the central section, the central to the central-posterior sections, as well as the central-posterior to the posterior section (*p*s > .113). On the right side, the gradient was observed from the frontocentral to the central section and the central to the central-posterior section (*p*s > .056).

In the extension_time-window_ analysis (*F*(8,832) = 8.29, *p* < .001, $\eta_{p}^{2}$= .07, GG = .49), the anterior gradient was there except on the left side for the differences between the frontal and frontocentral section and the frontocentral and central section (*p*s > .36).

In the extension_CSD_ analysis (*F*(8,832) = 39.04, *p* < .001, $\eta_{p}^{2}$= .27, GG = .37), for the left and right sites, the P3 signal was more positive on posterior positions (*p*s < .001), followed by the central posterior position (*p*s < .05), except on the left sites were the frontal position was not different (*p* = .275). On the right sites, the frontal position exhibited more positive values than the frontocentral electrodes (*p* < .001). For the midline electrodes, the central-posterior sites had the most positive signals (*p*s < .001), followed by posterior and central positions (*p*s < .001). The most negative values were observed at frontal and frontocentral positions (*p*s < .001).

#### Paradigm x Anteriority

Concerning the paradigm x anteriority interaction, post-hoc tests in the replication analysis (*F*(4,416) = 3.76, *p* < .01, $\eta_{p}^{2}$= .04, GG = .29) revealed that the anterior gradient was present in the UG (*p*s < .01). However, it was not complete for the DG (*p*s < .05), showing the same values for the frontal and frontocentral section, the frontocentral and central section, and the central and central-posterior section (*p*s > .20).

There was no significant interaction effect for extension_time-window_. Yet, for extension_CSD_ (*F*(4,416) = 4.79, *p* < .001, $\eta_{p}^{2}$= .04, GG = .53), post-hoc tests indicated that the most positive signals were present on central-posterior and posterior sites (*p*s < .001), except for the DG where the posterior signal was lower (*p* < .001). For the UG, the signals on the central sites were also more positive than on frontal and frontocentral sites (*p*s < .001).

#### Paradigm x Laterality

For the interaction lateral x paradigm in the replication (*F*(2,208) = 8.85, *p* < .001, $\eta_{p}^{2}$= .08, GG = .94) and extension_time-window_ analyses (*F*(2,208) = 6.54, *p* < .01, $\eta_{p}^{2}$= .06, GG = .97), post-hoc tests indicated differences between the left, right, and midline sites for the UG (*p*s < .001). For the DG, only the difference between the left and right side was significant in the replication (*p* = .017). There was no such difference in the extension_time-window_ analysis (*p*s > .19).

In the extension_CSD_ analysis (*F*(2,208) = 4.74, *p* < .01, $\eta_{p}^{2}$= .04, GG = .83), post-hoc tests indicated that the signal was most positive on midline sites (*p*s < .001). Additionally, for the UG, the right sites were more positive than the left sites (*p* < .001).

#### Paradigm x Anteriority x Laterality

In the replication analysis (*F*(8,832) = 3.75, *p* < .001, $\eta_{p}^{2}$= .04, GG = .43), the three-way interaction between paradigm, the anterior factors, and the laterality factors revealed a gradient. The most prominent P3 posterior was observed on the left side in the UG (*p*s < .01). On the right side and the midline in the UG, as well as on both the right and left side in the DG, we only found differences to all other areas except the central-posterior position (*p*s < .05). In addition, the P3 signal was higher in the central-posterior position compared to the frontocentral and frontal regions on every side in the UG (*p*s < .01), while in the DG, only the frontal positions on the right and midline sites showed more negative P3 than the central-posterior sites (*p*s < .01). Moreover, there was a significant difference between the midline frontocentral and the midline frontal area in the UG (*p* < .001).

In the extension_time-window_ analysis (*F*(8,832) = 3.17, *p* < .01, $\eta_{p}^{2}$= .03, GG = .42, see Figure S11b panel A), the gradient showed the most prominent P3 posterior in the UG (*p*s < .05), except for a lack of difference between a frontal and frontocentral electrode (*p*s > .811) and between the central and frontocentral electrodes on the right and left sites (*p*s = 1). For the DG, the highest P3 was found posterior (*p*s < .01), apart from midline sites, where the posterior and the central-posterior electrodes did not differ (*p* = 1). In addition, the central-posterior electrodes showed higher P3 amplitudes than relatively more frontal electrodes (*p*s < .05) except on the lateral sites, where no significant difference was found between the central-posterior and central electrodes (*p*s > .10). Furthermore, for the right and midline sites, significant differences were observed between frontal and central electrodes (*p*s < .01)

In the extension_CSD_ (*F*(8,832) = 4.79, *p* < .001, $\eta_{p}^{2}$= .04, GG = .41; see Figure S11b panel B), we identified significantly higher P3 amplitudes on posterior sites (*p*s < .001), except for the midline sites, where the most positive values were shown at central-posterior sites (*p*s < .001), while posterior and central sites did not differ. These sites were in turn more positive than the frontal and frontocentral sites (*p*s < .001). On the right side, central-posterior sites were more positive than frontocentral and central sites (*p*s < .05). On the left side, this difference was only present for the UG and the central sites (*p* < .01).


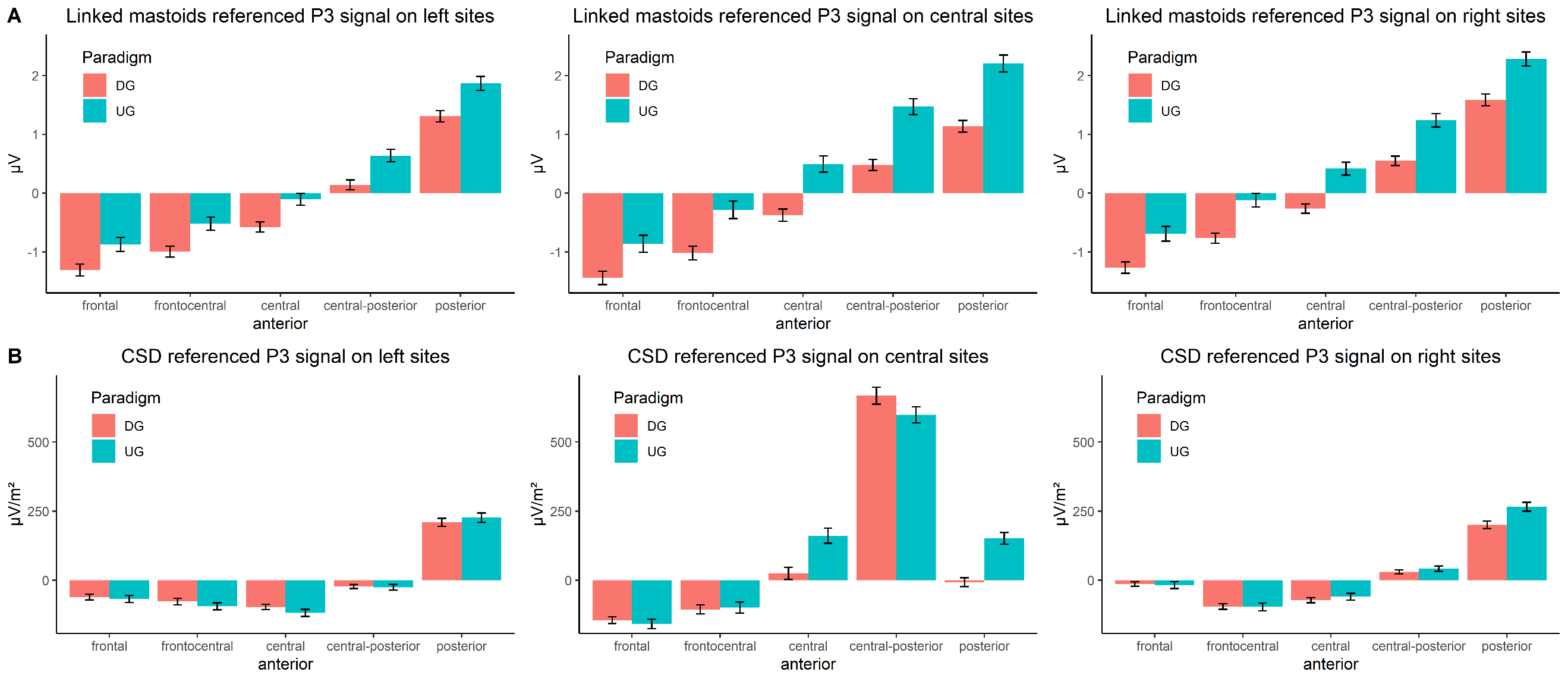


FigureS11b: A. P3 threefold interaction between paradigm, anteriority, and laterality using the new time window quantification with linked mastoid reference. B. The same interaction using the new time window quantification with CSD reference. Error-bars depict the mean.

#### Offer x Anteriority x Laterality

In the replication analysis (*F*(40,4160) = 2.89, *p* < .001, $\eta_{p}^{2}$= .03, GG = .38), the threefold interaction offer x anterior x lateral could not be interpreted using post-hoc *t-*tests as all offer differences became non-significant (*p*s > .25), possibly due to the overly conservative correction of the Bonferroni-Holm method.

For the extension_time-window_ (*F*(40,4160) = 5.39, *p* < .001, $\eta_{p}^{2}$= .05, GG = .35), the only significant difference was observed at the midline frontal electrode for the offer 1 vs. offer 4 (*p* = .024; see Figure S11c panel A).

For the extension_CSD_ (*F*(40,4160) = 7.73, *p* < .001, $\eta_{p}^{2}$= .07, GG = .37), we identified a difference at the midline central-posterior electrode for the 5 and 6-credits offers, showing more negative values than the 1, 2, and 3-credits offers (*p*s < .001). In addition, on this position, the 4-credits offer was more negative than the highest offer (*p* < .05) and the 2-credits offer (*p* < .01; see Figure S11c panel B).


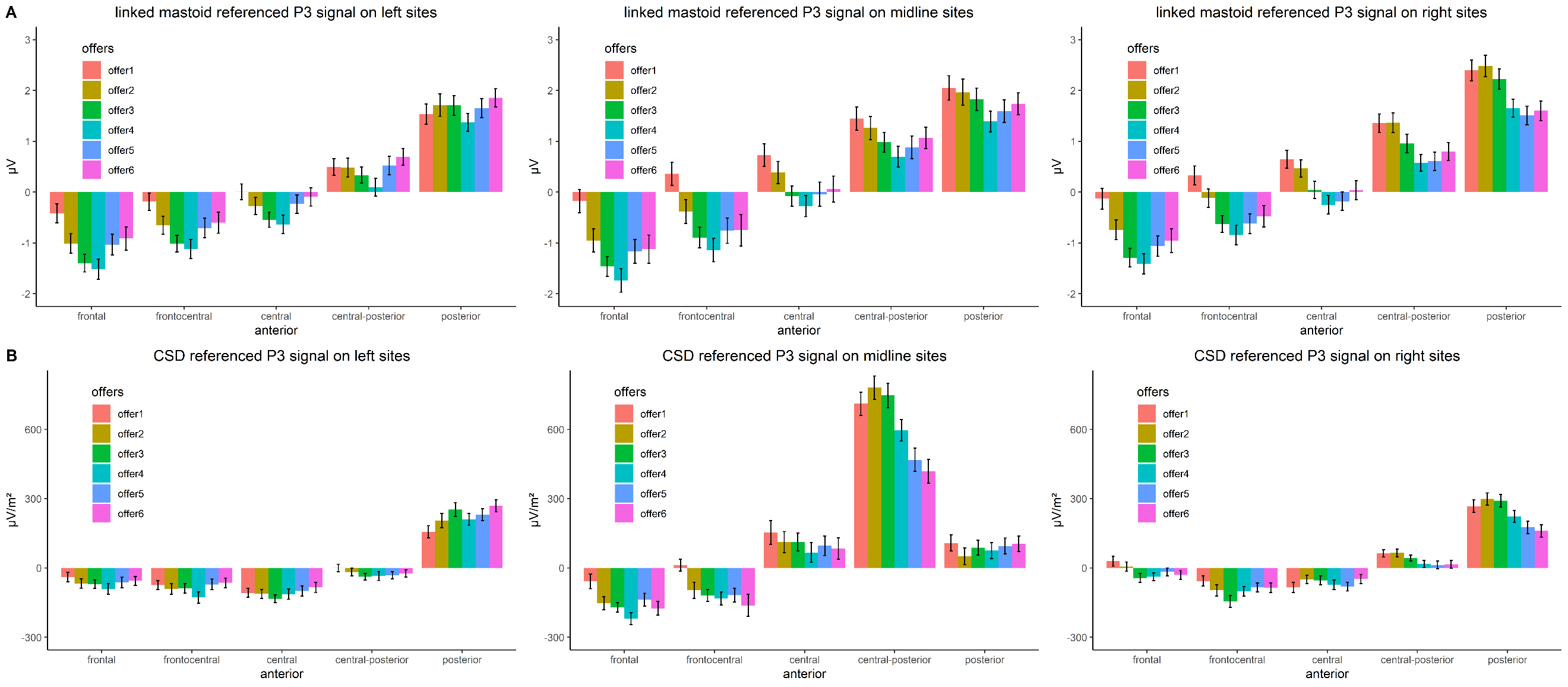


Figure S11c: A. P3 threefold interaction offer, anteriority, and laterality, using the new time window quantification with linked mastoid reference. B. The same interaction using the new time window quantification with CSD reference. Error-bars depict the mean within SEM.
